# Supplementary material for: The Polycomb-Dependent Epigenome Controls β Cell Dysfunction, Dedifferentiation, and Diabetes
Source: Cell Metab. 2018 Jun 5;27(6):1294–1308.e7. doi: 10.1016/j.cmet.2018.04.013 (PMC5989056; doi:10.1016/j.cmet.2018.04.013)
Supplement: Document S1. Figures S1–S5 [file mmc1.pdf]

Cell Metabolism, Volume *27*

Supplemental Information

The Polycomb-Dependent Epigenome Controls β Cell Dysfunction, Dedifferentiation, and Diabetes

Tess Tsai-Hsiu Lu, Steffen Heyne, Erez Dror, Eduard Casas, Laura Leonhardt, Thorina Boenke, Chih-Hsiang Yang, Sagar, Laura Arrigoni, Kevin Dalgaard, Raffaele Teperino, Lennart Enders, Madhan Selvaraj, Marius Ruf, Sunil J. Raja, Huafeng Xie, Ulrike Boenisch, Stuart H. Orkin, Francis C. Lynn, Brad G. Hoffman, Dominic Grün, Tanya Vavouri, Adelheid M. Lempradl, and J. Andrew Pospisilik

Figure S1 (Related to Figure 1)

A

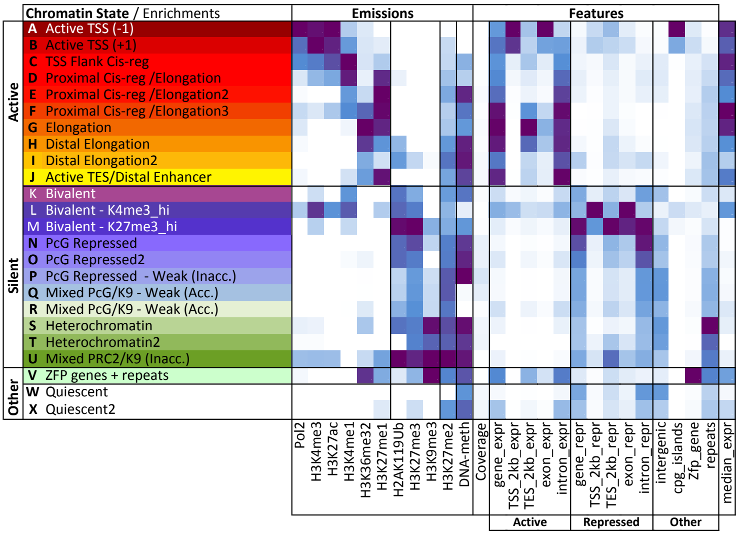

B

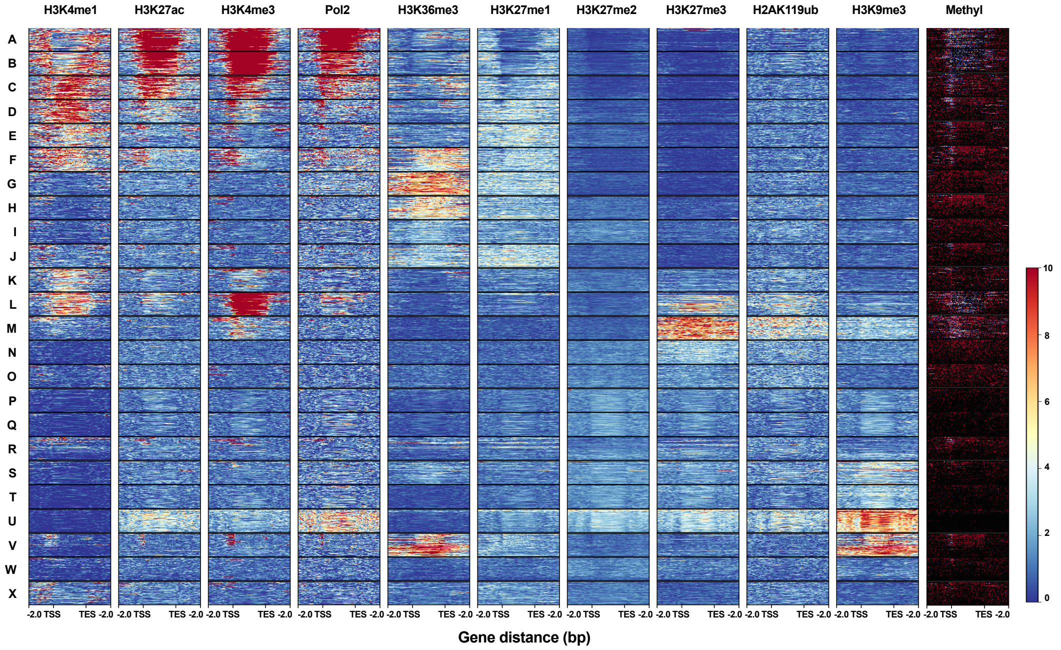

C

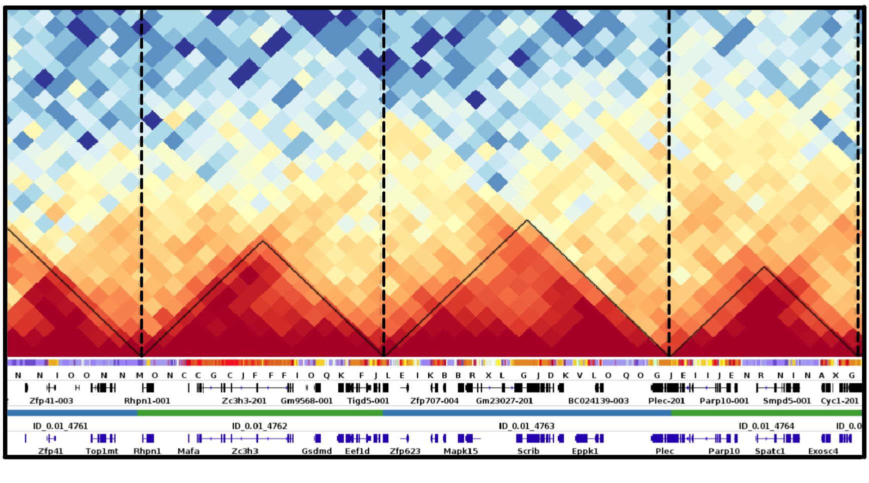

Supplementary Figure S1.

A.Histone mark characteristics, gene expression levels and genomic features of healthy islets. EpiCSeq chromatin state segmentation and manual annotation according to emissions and annotations in D. (Inacc=Inaccessible; +1 or -1=± nucleosome position around TSS, expr = expressed, repr= repressed, median_expr= Median expression)

B.Heatmap profiles of average normalized read coverage from ChIP-seq signals for various histone marks, Pol2 and CpG methylation signal of 100 genes from each chromatin state.

C.Example region showing Hi-C TAD boundaries aligning with chromatin state segmentation boundaries. Dash lines indicates TAD boundaries. (Hi-C image taken from http://chorogenome.ie-freiburg.mpg.de; dataset mouse CH12; GEO accession GSE63525)

Figure S2 (Related to Figure 2)

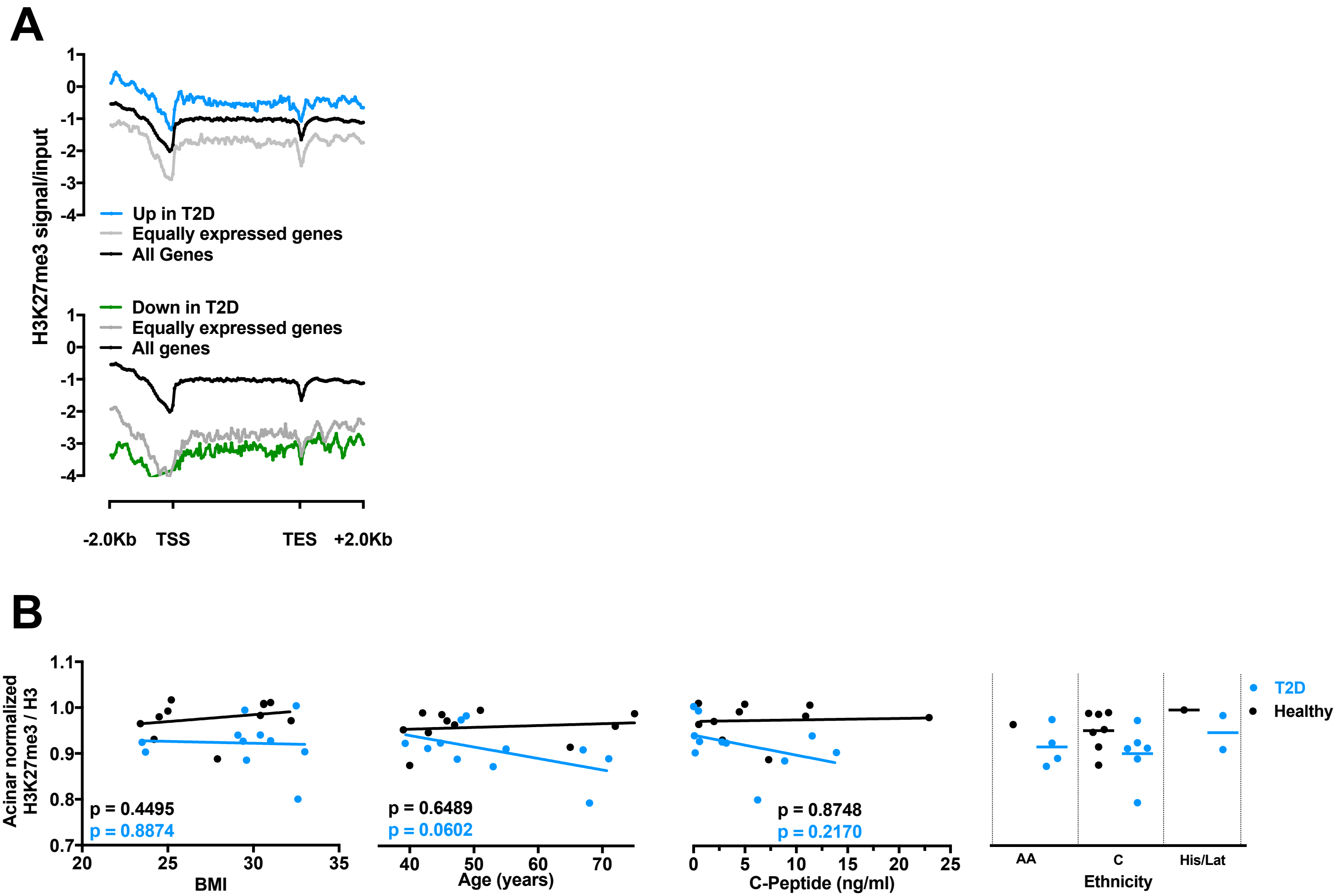

Supplementary Figure S2.

A. Average H3K27me3 signals at genes up or down-regulated in T2D and equally expressed genes. TSS: transcription start site, TES: transcription end.

B. H3K27me3 immunostaining intensity in β-cells of pancreatic sections from T2D healthy (black) and diabetic (blue) donors vs BMI, age, C-peptide levels or ethnicity. Solid lines = linear regression analysis of groups. $P < 0.05$ indicates regression slopes being significantly non-zero.

Supplementary Figure 3 (Related to Figure 3)

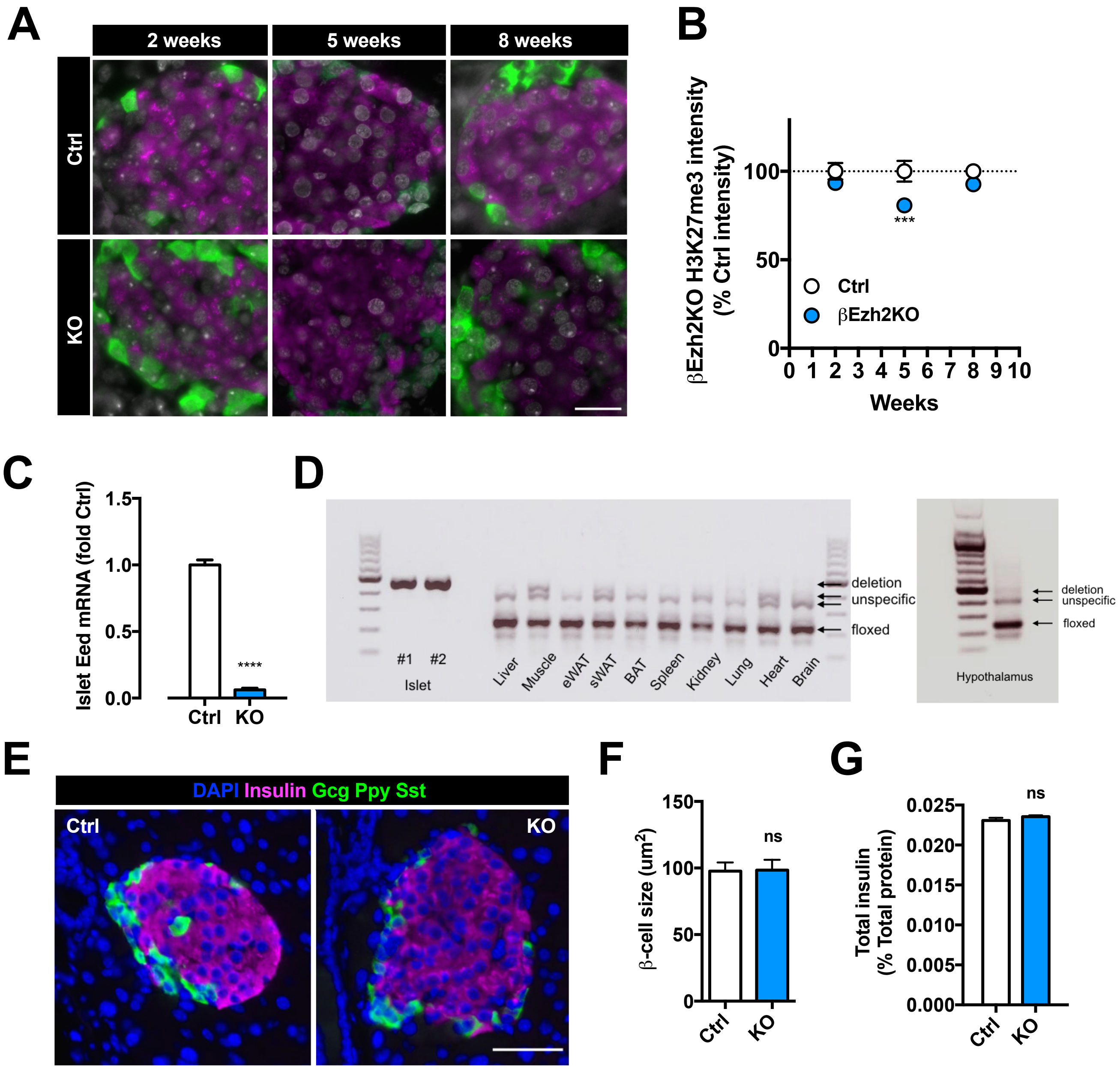

Supplementary Figure S3

A.Representative images of immunostaining for H3K27me3 (grey), insulin (magenta) and glucagon (green) in Ctrl (top) and βEzh2KO (bottom) islets at 2, 5 and 8 weeks of age.

B.Quantification of relative intensity of H3K27me3 immunostainings of βEedKO and control islet β-cells at different ages. All data points were normalized to alpha-cell H3K27me3 levels in the same islets prior to comparison.

C.Quantitative RT-PCR showing loss of Eed mRNA in βEedKO islets.

D.PCR validation of *Eed* allele deletion in islets and other tissues of βEedKO animals. One animal is represented here for all tissues except for islets where two animals are shown

E.Immunofluorescence staining of islet hormones in Ctrl and βEedKO islets at 8 weeks of age.

F.-G. Quantification of total β-cell mass (left) and insulin area (right) per islet shows no significant difference between Ctrl and βEedKO animals at 8 weeks of age.

Data represents mean ± SEM. Scale bar = 25uM. *** $p<0.001$, **** $p<0.0001$, ns = $p>0.05$.

Supplementary Figure 4 (Related to Figure 4)

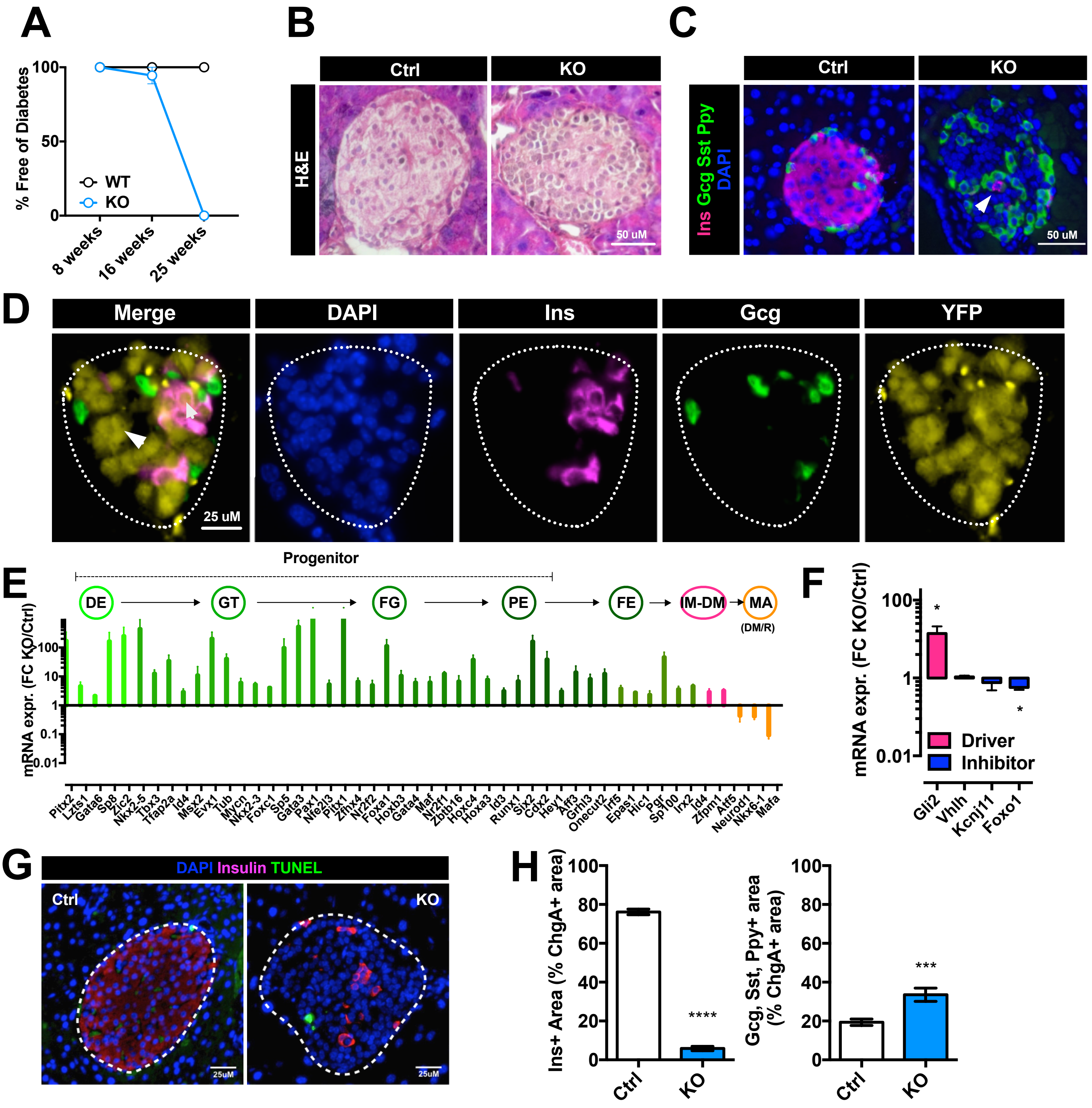

Supplementary Figure S4.

A.Percentage of diabetes-free individuals at 8, 16 and 25 weeks of age. Diabetes was defined as blood glucose >300mg/dL after 2 hours fasting. Note that all animals developed diabetes at 25 weeks of age

B.H&E staining of pancreatic sections showing readily detectable islets and reduced inter-nuclei spacing in βEedKO mice

C.Immunofluorescence stainings for hormones of ctrl and βEedKO islets. White arrow depicts cell with residual insulin staining.

D.Representative image of βEedKO islets harbouring a RIP-cre inducible YFP lineage tracer (yellow) stained for insulin (magenta) and glucagon (green). Small white arrow indicates insulin and YFP positive β-cell, large white arrow indicates insulin negative YFP positive cell. White dotted line outlines the islet.

E.Mean expression fold change of transcription factors in mRNA-seq data from βEedKO and Ctrl islets (Gene sets from DE: definitive endoderm, GT: gut tube, FG: fore gut, PE: pancreatic endoderm; Xie et al., 2013) and immature β-cells (IM-DM; Blum et al., 2012, MA: REACTOME: *Regulation of Gene Expression in Beta-Cell*). Arrow represent hypothetical developmental trajectory. Genes listed show core enrichment in GSEA analysis and are significantly different between healthy and T2D islets with FDR-q<0.05.

F.Mean expression fold change of known factors implicated in β-cell de-differentiation in mRNA-seq data from βEedKO and Ctrl islets. * FDR-q<0.05

G.Representative images of TUNEL treated Ctrl and βEedKO pancreas. White dotted line indicates the border of islets

H.Morphometric analysis of immunostaining: % β-cell area (Ins+, left panel) and α- (Gcg+) , δ- (Sst+) and PP-cell (Ppy+) area (right panel) within the endocrine compartments (ChgA positive) of islets at 25 weeks of age. *** p<0.001, **** p<0.0001

Figure S5 (Related to Figure 5)

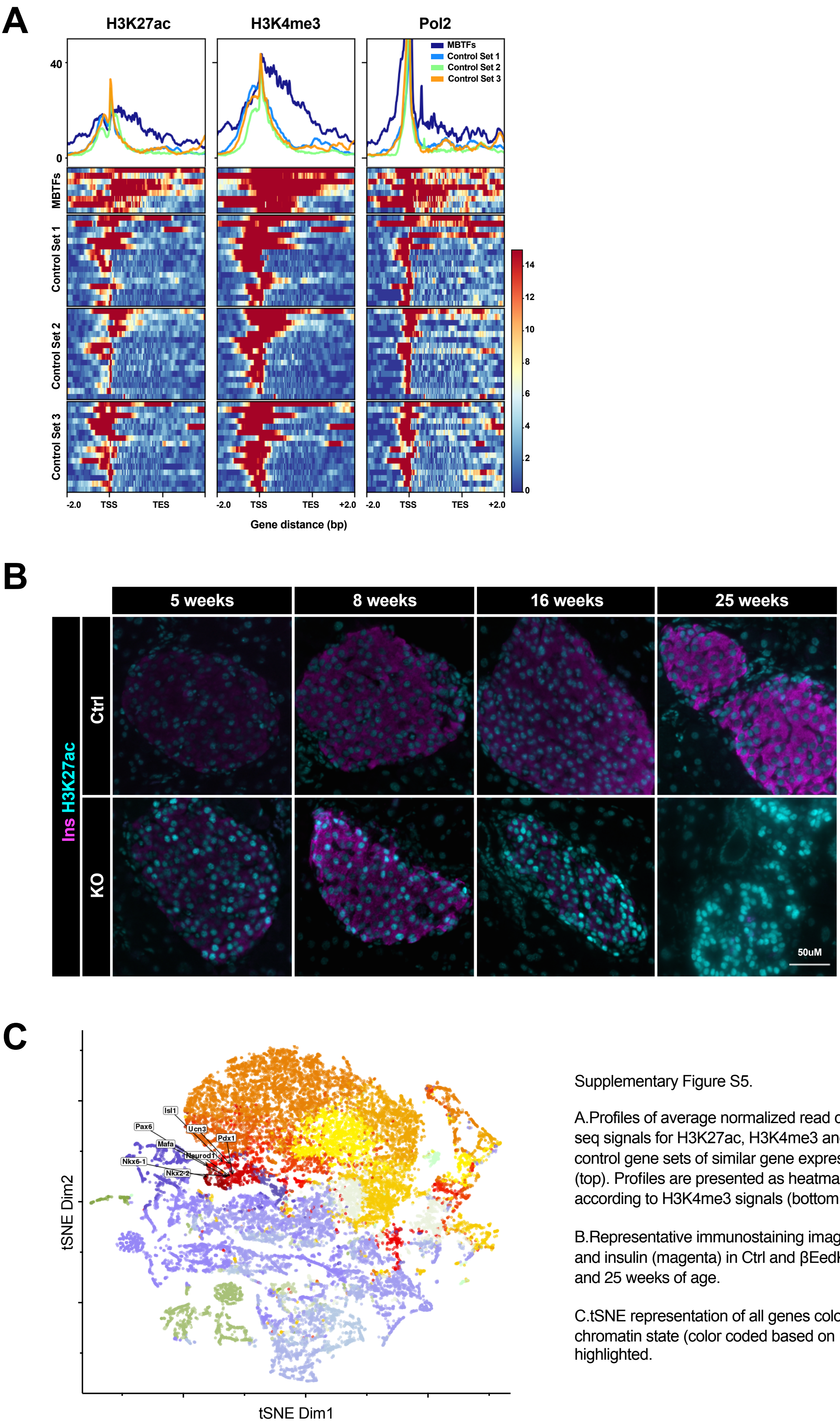

Supplementary Figure S5.

A.Profiles of average normalized read coverage from ChIP-seq signals for H3K27ac, H3K4me3 and PolII for MβTFs and control gene sets of similar gene expression in mouse β-cells (top). Profiles are presented as heatmaps and sorted according to H3K4me3 signals (bottom).

B.Representative immunostaining images for H3K27ac (cyan) and insulin (magenta) in Ctrl and βEedKO islets at 5, 8, 16, and 25 weeks of age.

C.tSNE representation of all genes colored according to chromatin state (color coded based on Figure 1E). MβTFs are highlighted.
