# Supplementary material for: The Polycomb-Dependent Epigenome Controls β Cell Dysfunction, Dedifferentiation, and Diabetes
Source: Cell Metab. 2018 Jun 5;27(6):1294–1308.e7. doi: 10.1016/j.cmet.2018.04.013 (PMC5989056; doi:10.1016/j.cmet.2018.04.013)
Supplement: Document S2. Article plus Supplemental Information [file mmc7.pdf]

Cell Metabolism

Article

The Polycomb-Dependent Epigenome Controls β Cell Dysfunction, Dedifferentiation, and Diabetes

Graphical Abstract

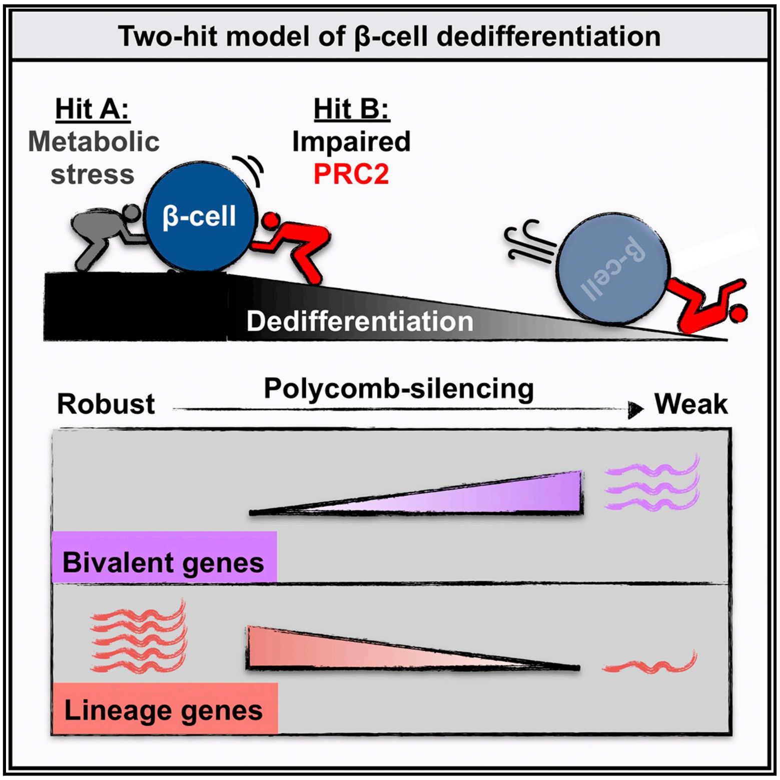

Authors

Tess Tsai-Hsiu Lu, Steffen Heyne, Erez Dror, ..., Tanya Vavouri, Adelheid M. Lempradl, J. Andrew Pospisilik

Correspondence

pospisilik@ie-freiburg.mpg.de

In Brief

Lu et al. provide evidence of chromatin dysregulation in type 2 diabetes in mice and humans. Loss of Polycomb silencing in mouse pancreas triggers hyperglycemia-independent dedifferentiation of β cells and diabetes, suggesting a two-hit (chromatin and hyperglycemia) model for loss of β cell identity in diabetes.

Highlights

- Deep epigenome mapping and single-cell transcriptomics of β cells in T2D
- Human and mouse diabetes mimic PRC2 loss of function
- Eed/PRC2 safeguards transcription integrity in terminally differentiated β cells
- Eed/PRC2-sensitive dedifferentiation is pharmacologically targetable

Lu et al., 2018, Cell Metabolism *27*, 1294–1308
June 5, 2018 © 2018 The Author(s). Published by Elsevier Inc.
https://doi.org/10.1016/j.cmet.2018.04.013

Check for updates

CellPress

Cell Metabolism
Article

The Polycomb-Dependent Epigenome Controls β Cell Dysfunction, Dedifferentiation, and Diabetes

Tess Tsai-Hsiu Lu,[1,7,9] Steffen Heyne,[1,9] Erez Dror,[1] Eduard Casas,[2] Laura Leonhardt,[1,6] Thorina Boenke,[1] Chih-Hsiang Yang,[1] Sagar,[1] Laura Arrigoni,[1] Kevin Dalgaard,[1,7] Raffaele Teperino,[1,8] Lennart Enders,[1] Madhan Selvaraj,[1,7] Marius Ruf,[1] Sunil J. Raja,[1] Huafeng Xie,[4] Ulrike Boenisch,[1] Stuart H. Orkin,[4] Francis C. Lynn,[5] Brad G. Hoffman,[5] Dominic Grün,[1] Tanya Vavouri,[2,3] Adelheid M. Lempradl,[1] and J. Andrew Pospisilik[1,10,*]
[1]Max Planck Institute of Immunobiology and Epigenetics, Stuebeweg 51, 79108 Freiburg, Germany
[2]Program for Predictive and Personalized Medicine of Cancer (PMPPC), Institute Germans Trias i Pujol, UAB, Badalona 08916, Spain
[3]Josep Carreras Leukaemia Research Institute (IJC), Campus ICO - Germans Trias i Pujol, Badalona 08916, Spain
[4]Department of Pediatric Oncology, Dana-Farber Cancer Institute and Division of Hematology/Oncology, Boston Children's Hospital, Harvard Stem Cell Institute, Harvard Medical School, Boston, MA 02115, USA
[5]Diabetes Research Program, BC Children's Hospital Research Institute, A4-184, 950 West 28 Avenue, Vancouver, BC V5Z 4H4, Canada
[6]Diabetes Center, Department of Medicine, University of California, San Francisco, San Francisco, CA 94143, USA
[7]Present address: Novo Nordisk A/S, Novo Nordisk Park, 2760 Måløv, Denmark
[8]Present address: Institute of Experimental Genetics, Helmholtz Zentrum Munich and German Center for Diabetes Research (DZD), 85764 Neuherberg, Germany
[9]These authors contributed equally
[10]Lead Contact
*Correspondence: pospisilik@ie-freiburg.mpg.de
https://doi.org/10.1016/j.cmet.2018.04.013

SUMMARY

To date, it remains largely unclear to what extent chromatin machinery contributes to the susceptibility and progression of complex diseases. Here, we combine deep epigenome mapping with single-cell transcriptomics to mine for evidence of chromatin dysregulation in type 2 diabetes. We find two chromatin-state signatures that track β cell dysfunction in mice and humans: ectopic activation of bivalent Polycomb-silenced domains and loss of expression at an epigenomically unique class of lineage-defining genes. β cell-specific Polycomb (Eed/PRC2) loss of function in mice triggers diabetes-mimicking transcriptional signatures and highly penetrant, hyperglycemia-independent dedifferentiation, indicating that PRC2 dysregulation contributes to disease. The work provides novel resources for exploring β cell transcriptional regulation and identifies PRC2 as necessary for long-term maintenance of β cell identity. Importantly, the data suggest a two-hit (chromatin and hyperglycemia) model for loss of β cell identity in diabetes.

INTRODUCTION

Complex diseases such as cancer, autoimmunity, obesity, and diabetes represent some of the greatest socio-economic challenges of our day. They result from genetic predisposition but contain equally strong non-genetic components, often termed "environmental influences," that alter susceptibility, reversibility, and triggering of disease. Non-genetic regulation is believed to converge upon chromatin-dependent processes, etiological contributions that remain poorly understood.

Diabetes affects more than 400 million individuals worldwide (WHO, 2016). It presents predominantly in either an early-onset autoimmune type 1 or a heterogeneous type 2 diabetes (T2D) associated with obesity, inflammation, and insulin resistance. Ultimately, diabetes results from insufficient insulin-secreting β cell mass, resulting from impaired function, increased cell death, or loss of cell identity. The last decades have seen major advances in our understanding of β cell identity, development, and plasticity. β Cells are believed to be highly plastic (Stanger and Hebrok, 2013; Ziv et al., 2013), and pioneering studies over the last decades have revealed networks of transcriptional and chromatin regulators that drive β cell lineage development (Oliver-Krasinski and Stoffers, 2008; Arda et al., 2013; van Arensbergen et al., 2010, 2013; Bramswig et al., 2013; Xu et al., 2011) and that provide barriers against transdifferentiation or loss of β cell identity (Talchai et al., 2012; Dhawan et al., 2011; Puri et al., 2013; Taylor et al., 2013; Gao et al., 2014; Papizan et al., 2011; Swisa et al., 2017; Ediger et al., 2017; Collombat et al., 2009; Gutiérrez et al., 2017).

To date, it remains poorly understood how transcriptional programs are stabilized over the long cellular lifespans that can be found *in vivo*. Since adult β cells are both long-lived and highly plastic, mechanisms are thought to be in place to continuously reinforce and stabilize the terminally differentiated state (Cnop et al., 2011; Szabat et al., 2012). Seminal studies in both wild-type (Guo et al., 2013; Laybutt et al., 2003) and in genetic (Talchai et al., 2012; Brereton et al., 2014; Wang et al., 2014) models have highlighted relative losses of β cell identity, or "dedifferentiation," with mounting metabolic stress. Dedifferentiation was

 © 2018 The Author(s). Published by Elsevier Inc.
This is an open access article under the CC BY license (http://creativecommons.org/licenses/by/4.0/).

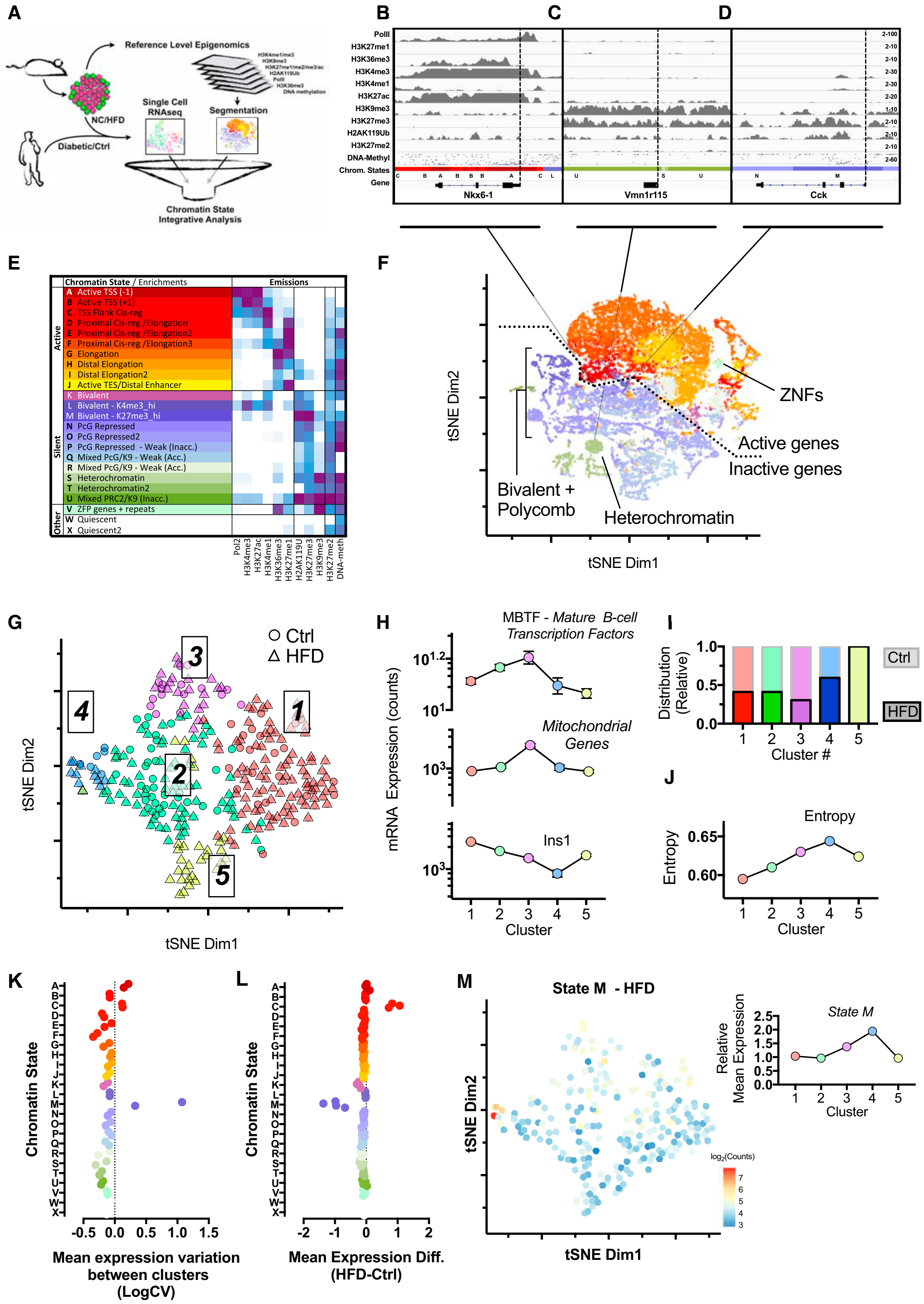

(legend on next page)

coined to describe *either* a reversal of the differentiation trajectory back toward progenitor states *or* a loss of terminal differentiation markers and phenotypes (Holmberg and Perlmann, 2012; Weir et al., 2013). Studies have documented the phenomenon in culture (Russ et al., 2008) and in T2D, in rodents and in humans tissues, and have focused on re-appearance of progenitor markers (ALDH1A; Cinti et al., 2016), as well as loss of lineage-defining gene expression as cardinal features (PDX1, MAFA, NKX6-1, INS, and GLUT2; Guo et al., 2013). To date, aside from identification of a limited number of inducers (hyperglycemia, β cell inexcitability, and NPAS4 or FoxO1 deficiency), we understand little of the molecular mechanisms that define how and when dedifferentiation occurs (Sabatini et al., 2018; Bensellam et al., 2017).

One chromatin-regulatory system important to defining cell fate trajectories is Polycomb. Polycomb comprises two sets of repressive complexes, PRC1 and PRC2, that mediate stable gene silencing through time and cell division (Margueron and Reinberg, 2011; Schuettengruber and Cavalli, 2009). PRC1 and PRC2 are non-redundant, with distinct loss-of-function phenotypes. PRC2 methylates the histone lysine residue H3K27 and is sufficient to silence gene expression (Margueron and Reinberg, 2011). PRC1 ubiquitinates H2AK119 at PRC2 marked domains, promoting chromatin compaction and further silencing (Simon and Kingston, 2013). Numerous PRC1 and PRC2 subcomplexes have emerged in recent literature, revealing additional unexplored complexities. Redundancies also exist, a prime example being the core PRC2 methyltransferases themselves, Ezh1 and Ezh2 (Xie et al., 2014; Ezhkova et al., 2011).

Here, we used unbiased epigenome mapping and single-cell RNA sequencing (scRNA-seq) to explore the chromatin dependence of transcriptional regulation in β cells. We observed two signatures of chromatin-state-associated transcriptional dysregulation consistent between human T2D- and high-fat diet (HFD)-driven β cell dysfunction: first, a loss-of-silencing at poised/bivalent Polycomb domains, and, second, collapse of gene expression at a unique subset of highly accessible active domains including cardinal lineage determinants. β cell-specific loss of Eed/PRC2 not only recapitulated these key chromatin-state-associated changes, but also triggered highly penetrant, largely hyperglycemia-independent, β cell dedifferentiation, implicating impaired PRC2 function as exacerbatory in diabetes. These findings identify Eed/PRC2 as necessary for maintenance of global gene silencing and terminal differentiation in β cells, and suggest a "two-hit" (chromatin and hyperglycemia) model of β cell dedifferentiation.

RESULTS

Chromatin-State-Specific Dysregulation Is a Hallmark of β Cell Dysfunction

To test for potential chromatin-driven regulatory events in β cell dysfunction we generated two orthogonal genomic analyses (Figure 1A). First, we used chromatin immunoprecipitation sequencing (ChIP-seq) to map high-dimensional epigenomes of mouse pancreatic β cells from healthy adult C57Bl6/J mice. We profiled histone marks characteristic for active and poised promoters (H3K4me3), enhancers (H3K27ac/H3K4me1), and transcribed coding regions (H3K36me3 and H3K27me1); heterochromatic- and Polycomb-silenced domains (H3K9me3 and H3K27me3/H2AK119Ub, respectively); quiescent intergenic regions (H3K27me2); transcription and accessibility (RNA-pol2); and complemented these with measurements of DNA methylation, an epigenetic mark which correlates depending on context with transcription, accessibility, CG-density, and/or promoter-silencing (WGBS; Avrahami et al., 2015). This extensive dataset provides in-depth genome-wide information on the nature of chromatin and transcriptional state in β cells, including at *cis*-regulatory elements, highly active loci (e.g., Nkx6-1; Figure 1B), silent genes (e.g., Vmn1r115; Figure 1C), and loci poised for transcriptional activation or repression (e.g., Cck; Figure 1D). These data represent a reference-level epigenome and a powerful resource for the community.

To simplify these high-dimensional data for flexible use with genome-wide association studies or transcriptome profiling, we performed chromatin-state segmentation, a dimensional reduction technique that provides every bin of the genome a single classification summarizing the many chromatin attributes of the locus (Figures 1E and 1F and colored bar in Figures 1B–1D, S1A, and S1B) (Mammana and Chung, 2015). Our 25-state model faithfully partitioned all ~25,000 genes into a range of transcribed and quiescent compartments and thus established a flexible and unbiased platform for exploring β cell chromatin function. Visualization by tSNE (Figure 1F; Table S1) provides

Figure 1. Chromatin State-Specific Dysregulation Is a Hallmark of β Cell Dysfunction

(A) Schematic of the generation, intersection, and analyses of chromatin driven regulatory events in β cell dysfunction.

(B–D) Histone mark occupancy and DNA methylation state at (B). Nkx6-1 (state A), (C) Vmn1r115 (state S), and (D) Cck (state M) gene loci. Dashed line indicates transcription start site (TSS).

(E) EpiCSeq chromatin-state segmentation in healthy islets and manual annotation according to emissions and annotations in D (Inacc, inaccessible; +1 or −1, ± nucleosome position around TSS).

(F) tSNE representation of all genes highlighting chromatin states in annotated colors based on (E). Dotted line segregates active and inactive genes. Solid lines point toward the epigenomic cluster of Nkx6-1, Vmn1r115, and Cck genes, respectively.

(G) tSNE representation of control diet (Ctrl) and high-fat diet (HFD) β cell transcriptomes clustering, indicating five distinct β cell sub-types.

(H) Average expression of MBTFs (top), mitochondrial genes (middle), and insulin (Ins1, bottom) from clusters 1–5 from (G).

(I) Relative fraction of cells across clusters 1–5 from (G) from either control (Ctrl) or high-fat diet (HFD)-treated samples.

(J) Median entropy level across clusters 1–5 from (G).

(K) Variation in mean gene expression in cluster 4 (Ctrl) and 5 (HFD) for all chromatin states. CV, coefficient of variation.

(L) Difference in mean gene expression between HFD and Ctrl for each of clusters 1–4 for all chromatin states.

(M) tSNE representation of mean expression levels of state M genes in HFD β cell transcriptomes (left panel). Relative mean expression of state M genes across clusters 1–5 (right panel).

See also Figure S1.

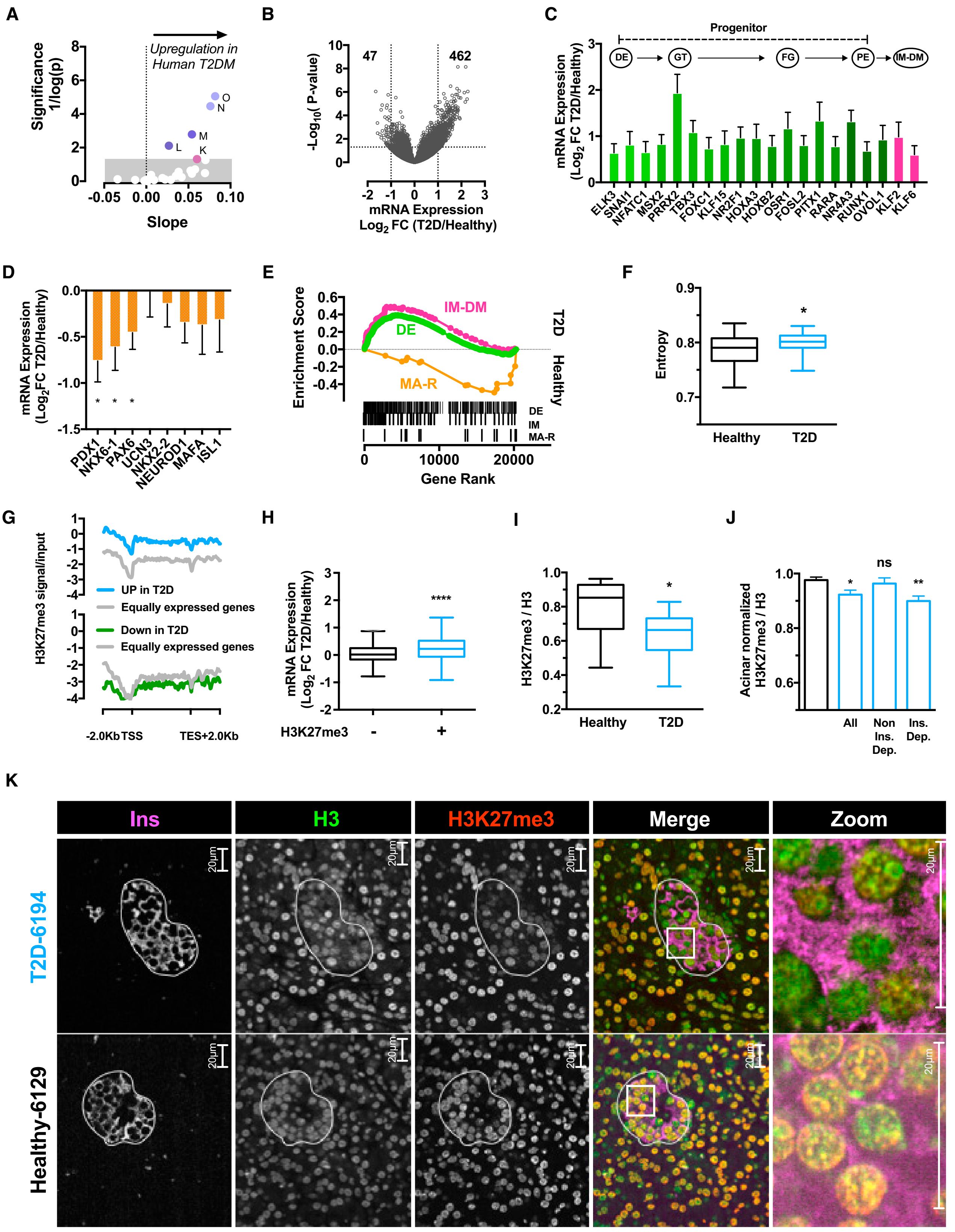

(legend on next page)

an unprecedented view of the heterogeneity of gene expression architecture in the β cell nucleus, highlighting a range of chromatic and transcriptional architectures at genes, *cis*-regulatory elements (Figure S1A), as well as megabase-scale spatial organization (Figure S1C). Importantly, the segmentation for the first time incorporates DNA methylation, extending the utility of these algorithms. Thus, we generated an integrated β cell epigenome useful for unbiased exploration by all β cell biologists.

In parallel, we used scRNA-seq to characterize the dynamics of β cell transcriptome heterogeneity in health and disease. Using a UMI- and 384-well fluorescence-activated cell sorting-based CELseq2 pipeline, we generated β cell transcriptomes from chow (NCD) and age-matched HFD-treated (18 weeks) C57Bl6/J mice (Figures 1G–1J). After normalization and filtering, the dataset included ~300 cells with a median ~15,000 unique transcripts per cell. Transcriptome clustering revealed five major β cell sub-states or -types (Figure 1G). The majority of cells fell into two of these characterized by robust mature β cell transcription factor (MβTF: Pdx1, Nkx6-1, Nkx2-2, Mafa, Pax6, Neurod1, Isl1, and Ucn3) and insulin gene expression (clusters 1 and 2; Figures 1G and 1H). Cells in cluster 3 were consistent with recently described "hub cells," exhibiting moderate insulin levels and high mitochondrial gene expression (Johnston et al., 2016). Clusters 4 and 5 contained fewer cells and exhibited low maturity and functional marker expression (Figures 1G and 1H). Importantly, the latter were primarily derived from HFD-challenged mice, demonstrating that HFD is sufficient to bias the islet cellular distribution toward dysfunctional or dedifferentiated β cell states (Figures 1G–1I). Importantly, careful examination revealed that cells from control islets also populated the poorly differentiated cluster 4 (7%; 6 out of 78 control cells) (Figure 1I), demonstrating that healthy islets also contain β cells, that exist at least transiently, in lowly differentiated states. Thus, we provide a platform for exploring the heterogeneity of β cell transcriptome dysregulation, and show that HFD is sufficient to bias the β cell population toward poorly differentiated cell states *in vivo*.

Finally, we intersected the two datasets. We grouped all genes by chromatin state and looked for abnormal variation across the five β cell clusters (Figures 1K–1M). Overall, detectable gene expression showed minimal variation across most chromatin states (Figure 1K). Intriguingly, though, genes embedded in three states (Figure 1E, states A, C, and M) did show signatures of chromatin-state-associated dysregulation. The most striking variation was observed for state M, which differed significantly between HFD and control cells within the same clusters (Figure 1L), as well as between the different β cell sub-types in each of the control or HFD datasets (Figure 1K). Chromatin state M loci were "bivalent" (H3K4me3 and H3K27me3 marked; typically thought of as poised for stable activation or repression) and characterized by high accessibility (DNA methylation low) and transcriptional quiescence (Pol2 and RNA-seq negative) (Figures 1D and 1E). State M genes increased with HFD and highly expressed genes such as insulin were reduced, suggesting elevated transcriptional entropy. High entropy is a transcriptome characteristic that tracks with "stemness" as stem cells lack highly active, cell-type-specific expression programs. Indeed, calculation revealed increased transcriptome-wide entropy in clusters 4 and 5 and indicated cluster 4 as the most dedifferentiated by the unbiased genome-wide measure (Figures 1J and 1M) (Grün et al., 2016). These data corroborate the reductions in maturity and functional β cell genes (Figure 1H). Thus, HFD triggers chromatin-state-defined transcriptome dysregulation and loss of cell identity in pancreatic β cells.

Polycomb (Dys)regulation and Dedifferentiation in Human T2D

To gauge relevance toward the human setting we examined islet RNA-seq data generated from 11 diabetic (HbA1c > 6.5) and 51 non-diabetic (HbA1c < 6.0) islet donors (Fadista et al., 2014). We ordered transcriptomes by HbA1c and examined ortholog expression according to our chromatin-state model (Figure 2A). Importantly, the results recapitulated the mouse analysis with genes in the bivalent state M (as well as the most closely related Polycomb states L, N, and O) upregulated in T2D patients (Figure 2A). This finding was consistent with a ~10-fold bias toward gene upregulation (Figure 2B; 462 up- versus 47 downregulated). This state-specific derepression was characterized by enriched alternate lineage factor expression, including genes

Figure 2. Polycomb (Dys)regulation and Dedifferentiation in Human T2D

(A) Significance and correlation (slope) of human orthologs embedded in respective mouse chromatin states in human T2D states (Fadista et al., 2014). Arrow indicates direction of positive correlation with gene upregulation in human T2D tested with linear regression. Upper boundary of gray box indicates p = 0.05.

(B) Volcano plot of mRNA-seq data from human T2D islets. Horizontal dotted line indicates p = 0.05, vertical lines indicates ±2-fold difference. Number of significantly up- or downregulated genes are shown.

(C) Mean expression fold change of transcription factors in mRNA-seq data from human T2D islets (gene sets from DE, definitive endoderm; GT, gut tube; FG, fore gut; PE, pancreatic endoderm; Xie et al., 2013) and immature β cells (IM-DM; Blum et al., 2012). Arrows represent hypothetical developmental trajectory. Genes listed show core enrichment in gene set enrichment analysis (GSEA) analysis and are significantly different between healthy and T2D islets with false discovery rate (FDR)-q < 0.05.

(D) Mean expression fold change of MBTFs between human T2D and healthy islet transcriptomes. *p < 0.05.

(E) GSEA of definitive endoderm (DE) (Xie et al., 2013), immature (IM-DM) (Blum et al., 2012), and mature β cell genes (MA(R); REACTOME, regulation of gene expression in β cell) in human T2D islets. FDR-q < 0.05 for all gene sets shown.

(F) Median entropy in T2D islet versus healthy islet transcriptomes. Boxplot whiskers indicate min to max.

(G) Average H3K27me3 signal at genes up- or downregulated in T2D and equally expressed genes. TSS, transcription start site; TES, transcription end.

(H) Boxplot of gene expression changes at H3K27me3 marked (+) and non-marked (−) genes in human T2D islets.

(I and J) H3K27me3 immunostaining intensity in β cells of pancreatic sections from T2D donors (n = 11, blue) relative to healthy donors (n = 11, black), normalizing to H3 intensity from the same nuclei (I) and normalizing against same image acinar nuclei (J). T2D donors are combined (All) or stratified by insulin treatment dependency (Non Ins. Dep. or Ins. Dep.).

(K) Representative images of a pancreatic islet (outlined) from a healthy (bottom) and T2D (top) donor pancreas sections. Zoomed-in areas are outlined in boxes. All data represent mean ± SEM, unless otherwise stated. Boxplot whiskers indicate 1.5 interquartile range, unless otherwise stated. *p < 0.05, **p < 0.01, ****p < 0.0001. ns, not significant. See also Figure S2.

characteristic of earlier stages in β cell development (Figure 2C), as well as reduced expression of MβTFs (Figure 2D). Both findings were true on the gene and pathway levels (Figures 2C–2E; Table S2). Again, transcriptional entropy increased with loss of glucose control (Figure 2F). To rule out species-specific confounding variables we also intersected the expression profiles with available H3K27me3 and H3K4me3 ChIP-seq data from sorted human β cells (Bramswig et al., 2013). The analysis validated the above findings. Genes upregulated in T2D were preferentially H3K27me3 marked in healthy islets (Figures 2G, 2H, and S2A). Thus, inappropriate activation of Polycomb-silenced bivalent domains, loss of transcription at key MβTFs, and elevated transcriptional entropy are conserved features of β cell dysfunction in human.

To test whether these changes resulted from true loss of Polycomb function, we examined H3K27me3 levels as a surrogate for PRC2 activity in sections from 11 T2D and 11 control human pancreas donors (BMI- and age-matched from nPOD). Sections were stained in unison, imaged double-blind, and insulin, H3, and H3K27me3 quantified in >30,000 cells covering at least 9 independent images per donor using an unbiased CellProfiler pipeline. Intriguingly, H3K27me3 was consistently reduced in diabetic β cell nuclei (Figures 2I–2K). Differences were maintained whether normalizing H3K27me3 to H3 as an internal reference (Figure 2I and 2K, reduced red/yellow nuclear staining within the islet in T2D "Merge"), or to surrounding acinar cell nuclei (Figures 2J). Reductions in H3K27me3 were independent of BMI, age, C-peptide level, and donor ethnicity (Figure S2B). Insulin-dependent T2D donors exhibited more severe H3K27me3 depletion, suggesting an association with disease severity (Figure 2J). Thus, human T2D is characterized by a relative loss of PRC2 function.

Eed/PRC2 Deficiency Triggers Progressive β Cell Dedifferentiation and Diabetes

To define the consequences of loss of PRC2 silencing, we sought to generate β cell-specific PRC2-deficient mice. We first examined β cell-specific Ezh2 knockout (KO) mice (βEzh2KO) previously reported to exhibit moderate diabetes secondary to defective β cell proliferation (Chen et al., 2009). βEzh2KO animals were also mildly diabetic in our hands (not shown). Consistent with redundancy between Ezh1 and Ezh2 (Xie et al., 2014), and their co-expression in β cells throughout development and into adulthood (not shown), βEzh2KO animals showed partial loss of H3K27me3 only in the most rapidly dividing phase of islet and pancreatic growth (Figures S3A and S3B). The findings highlight βEzh2KO mice as a unique example of the long-term consequences of modest epigenetic dysregulation in early life. To abrogate all PRC2 activities, we next generated β cell-specific Eed KO mice (βEedKO; Figure 3A). Deletion of Eed disassembles both Ezh1- and Ezh2-containing complexes rendering both enzymes non-functional (Xie et al., 2014). βEedKO mice were born healthy, at Mendelian ratios, and indistinguishable from control (Ctrl) littermates. Efficient deletion was confirmed on the mRNA and DNA levels (Figures S3C and S3D). Complete loss of H3K27me2 and H3K27me3 immunoreactivity confirmed total functional deletion (Figure 3B). Thus, we generated β cell-specific Eed/PRC2 loss-of-function mice.

Islet morphology in 8-week-old βEedKO animals was normal (Figure S3E). To determine the time course of loss of function, we tracked H3K27me3 levels from late embryogenesis—when RIP-Cre becomes active—to adulthood. H3K27me3 at embryonic day (E) 14.5 and E17.5 was unremarkable (Figure 3C). After birth, however, β cell H3K27me3 decreased progressively with stark loss between 2 and 5 weeks (Figure 3C). H3K27me3 was lost in >90% of all insulin-positive cells by 8 weeks of age (Figures 3D and 3E). Importantly, over the same age range we found no evidence of altered β cell mass (Figure 3F), size, or pancreatic insulin content (Figures S3F and S3G). Thus, β cell Eed/PRC2 is dispensable for islet development and growth to adulthood.

To test the physiological consequences of β cell-specific PRC2 loss, we measured glucose tolerance at 8, 16, and 25 weeks of age. Eight-week-old βEedKO mice were glucose tolerant (Figure 4A, top) with a tendency toward insulin oversecretion (Figure 4A, bottom). At 16 weeks of age βEedKO animals exhibited marked glucose intolerance (Figure 4B, top) and, intriguingly, by 25 weeks 100% of all knockouts exhibited overt diabetes (Figure 4C, top), with reductions in both basal and glucose-stimulated insulin levels (Figures 4B and 4C, bottom). Without insulin-pellet supplementation all animals rapidly succumbed to the condition. This lethal insulin-dependent diabetes was highly synchronized, with onset between 20 and 25 weeks of age in males (Figure S4A; 2 hr fasting glucose >300 mg/dL) and ~40 weeks in females (not shown).

Islets from 25-week-old βEedKO mice were largely normal in size, morphology (Figure S4B), endocrine cell organization, and relative number. Intriguingly, the expected β cell majority of the islet was devoid of all major islet endocrine hormones including insulin (Figures 4D, 4E, and S4C). Consistent with onset of diabetes, βEedKO-driven loss of insulin immunoreactivity was synchronized and progressive between 8 and 25 weeks of age (Figure 4E). Hormone-negative βEedKO cells were all H3K27me3 negative, indicating that they originated from the Eed-KO β cell compartment (Figure 4D), a notion that was validated when intersecting the βEedKO model with a Cre-inducible enhanced yellow fluorescent protein (eYFP) lineage-tracing allele (Figures 4F, large arrow, and S4D). Thus, Eed/PRC2 is required for long-term maintenance of β cell function, insulin expression, and glucose tolerance *in vivo*.

βEedKO cells exhibited reduced levels of the mature β cell markers Pdx1, MafA, Nkx2-2, and Nkx6-1 (Figure 4G), and thus loss of β cell identity. Of interest given the profound phenotype, expression profiling revealed very high correlation between wild-type and βEedKO transcriptomes (R^2 > 0.95; 25 weeks), indicating that KO cells remained highly similar to wild-type β cells (Figure 4H). βEedKO islets downregulated mature β cell genes as defined by the Melton group (Mature-DM) and Reactome (Mature-R; regulation of gene expression in β cell) (Figures 4H and 4I; Table S3). At the same time, they upregulated immature β cell- and progenitor-specific genes (Figures 4H, 4I, and S4E) (Blum et al., 2012; Xie et al., 2013), indicating that the cells had only undergone reversal of very late, terminal differentiation or maturation. βEedKO islets increased expression of Gli2, a genetically validated driver of dedifferentiation (Figure S4F) (Landsman et al., 2011). They showed moderate reduction in FoxO1 and no marked regulation of Vhlh and Kcnj11—genes previously shown to buffer loss of mature identity (Figure S4F).

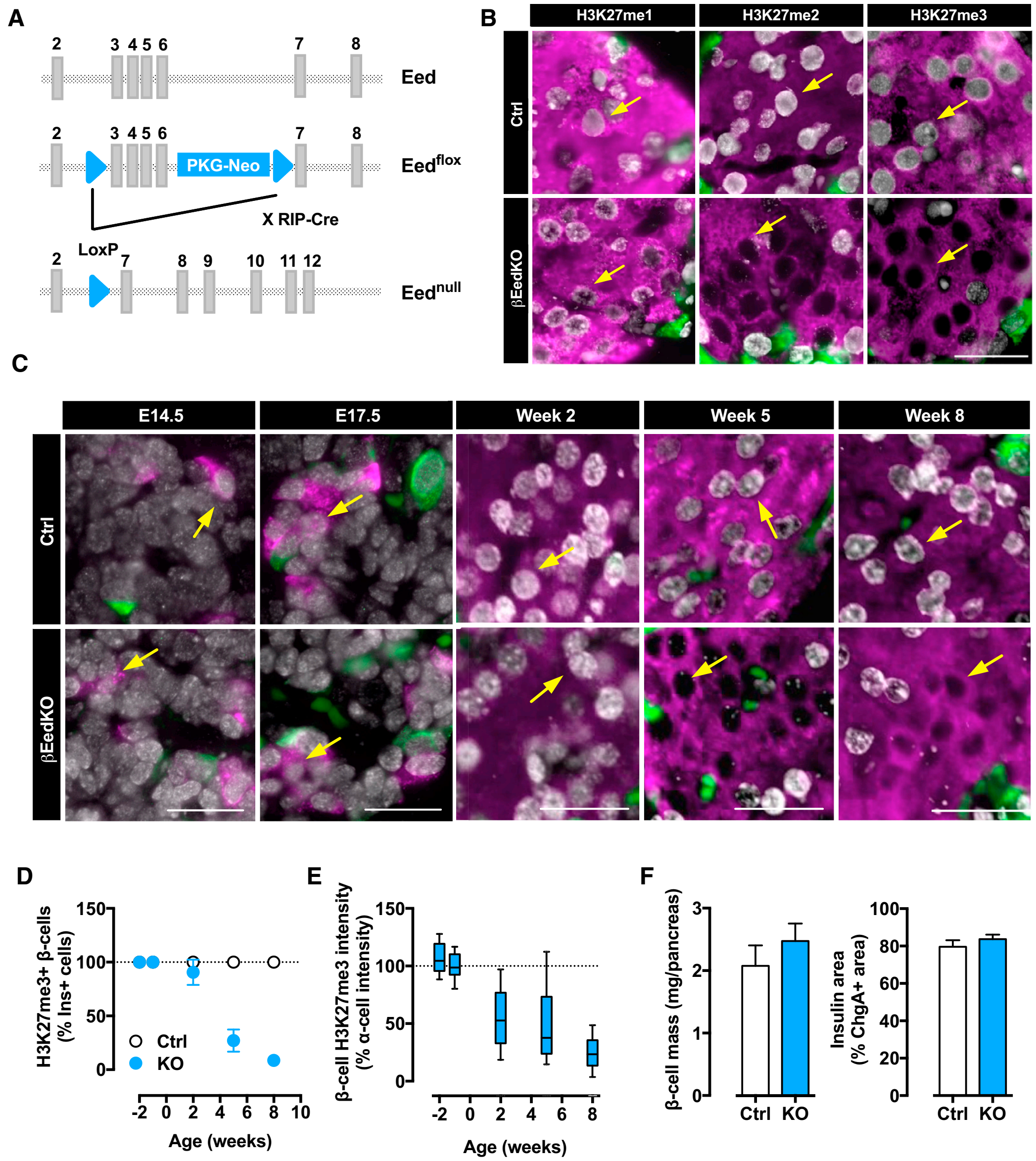

Figure 3. Eed/PRC2 Deficiency Triggers Progressive β Cell Dedifferentiation and Diabetes

(A) Schematic of the *Eed* targeting scheme. Light gray boxes depict exons (Xie et al., 2014).

(B) Immunofluorescence staining for H3K27me1, H3K27me2, and H3K27me3 (gray), insulin (magenta), and glucagon (green) in Ctrl and βEedKO. Yellow arrows indicate β cell nuclei.

(C) Representative images for H3K27me3 staining (gray) in Ctrl and βEedKO at the indicated ages. Insulin in magenta and glucagon in green. Yellow arrows indicate β cell nuclei.

(D) Quantification of H3K27me3-positive β cell number in pictures of βEedKO islets versus control immunofluorescence stainings.

(E) Mean β cell H3K27me3 fluorescence signals in βEedKO islets at different ages.

(F) Quantification of total β cell mass (left) and insulin area (right) per islet in Ctrl and βEedKO animals.

Data represent mean ± SEM of n = 4–5 mice per genotype for each experiment. Scale bars, 25 µM. See also Figure S3.

No reactivation was observed for the early progenitor markers Sox17, Oct4, Sox9, or Ngn3 (Puri et al., 2013; Talchai et al., 2012; Wang et al., 2014) (data not shown). No evidence was found of altered cell death (TUNEL; Figure S4G) or marked β to α cell transdifferentiation (by lineage tracing; not shown). Consistent with other diabetic models, we observed an ~15% increase in non-β cell mass (Figure S4H). Importantly, hormone-negative βEedKO cells retained expression of chromogranin A (Figure 4J), indicating maintained late endocrine character. Thus, loss of PRC2 function in β cells results in specific loss of terminal identity.

Seminal studies have highlighted hyperglycemia as one driver of β cell dedifferentiation (Bensellam et al., 2017). To test whether hyperglycemia contributed substantially to the βEedKO phenotype, we used subcutaneous insulin implants to normalize blood glucose long term in βEedKO mice, initiating treatment at

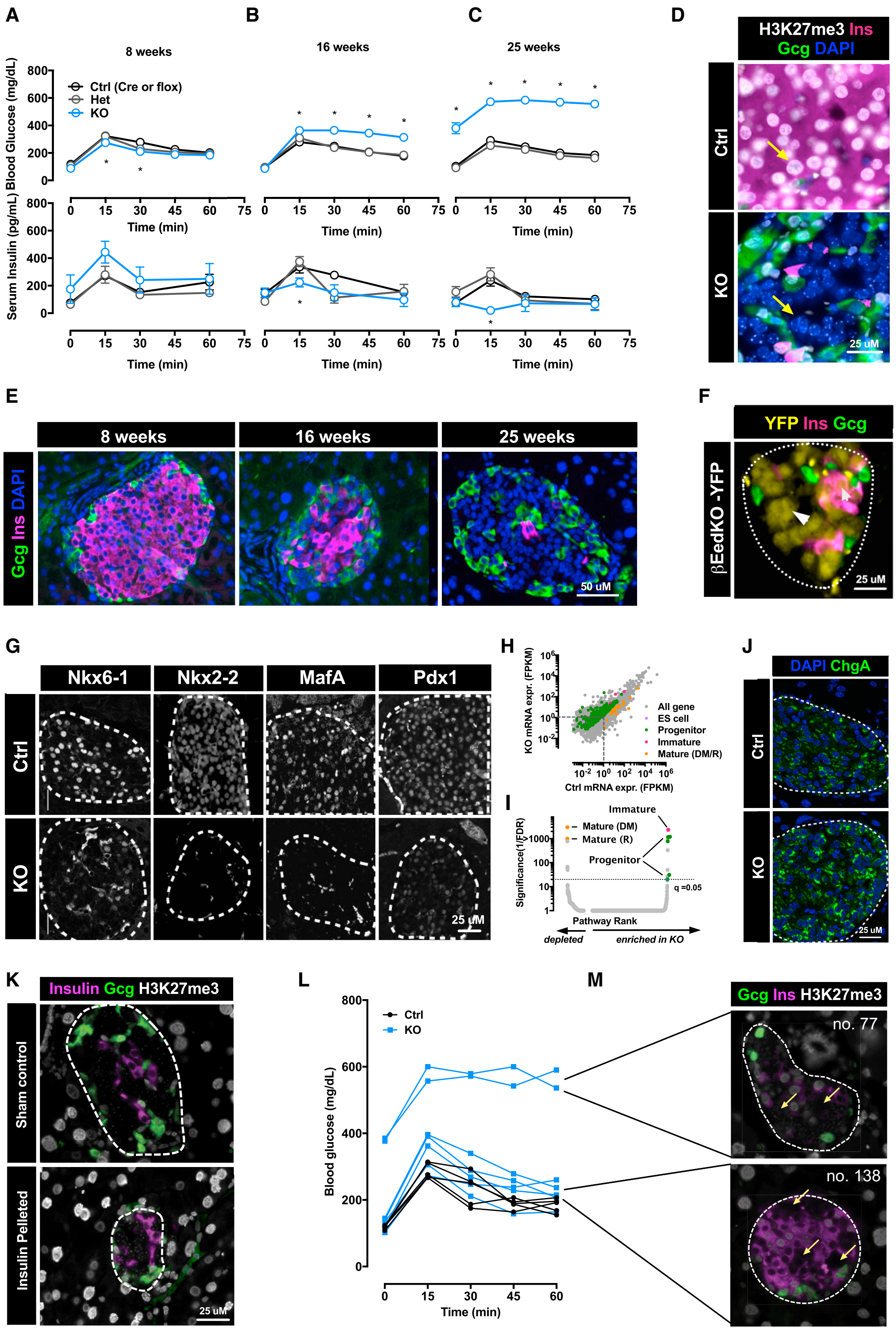

(legend on next page)

moderate (~18 weeks of age; normal fasting glucose) or late-stage pathology (~25 weeks; >90% dedifferentiation). Notably, these interventions failed to abrogate the EedKO-triggered dedifferentiation (Figure 4K) suggesting that hyperglycemia is not absolutely required for Polycomb-associated dedifferentiation. In a separate experiment, we took advantage of the low deletion efficiency of the Pdx1-Cre transgenic line to generate mice with mosaic Eed/PRC2 loss-of-function deletion in their islets. Pdx1-Cre; EedKO mice presented with a range of glycemic control from normoglycemic to moderate diabetes already at 12 weeks of age (Figure 4L). Importantly, all KO mice, including normoglycemic individuals, exhibited cells with robust loss of terminal differentiation. In all cases these cells were Eed/H3K27me3 deficient (Figure 4M). Together, these data indicate that PRC2-dependent dedifferentiation is cell autonomous and does not absolutely require dysglycemia. The data suggest that two largely independent axes contribute to dedifferentiation, one chromatin associated, and the other coupled to hyperglycemia. Thus, Eed/PRC2 is required for long-term maintenance of β cell identity *in vivo*.

Eed/PRC2 Maintains Global Silencing in Terminally Differentiated β Cells

Recapitulating the human and murine diabetes transcriptome data, we observed ~5-fold more up- than downregulation in βEedKO islets (Figure 5A; Table S4). These changes were progressive and evident already at 8 weeks of age, prior to hyperglycemia and identity loss. Whereas downregulation was observed primarily at active state A embedded genes (Figure 5B), upregulation was biased toward the bivalent Polycomb-silenced chromatin states L and M (DNA methylation low, transcriptionally quiescent, and H3K4me3/H3K27me3 double positive) (Figure 5B). Further, H3K27ac levels increased progressively after Eed KO (Figure S5B). H3K27ac ChIP-seq revealed ectopic H3K27ac deposition focused at >10,000 sharp novel peaks (Figure 5C). Ectopic peaks specifically appeared at pre-existing inactive but accessible promoters and enhancers (DNA methylation low loci marked by H3K4me1 and H3K4me3) (Figure 5D, gray shade). Consistent with *in vitro* work by Ferrari et al. (2014), our data support the notion that histone acetylation and methylation activities compete for H3K27 residues at chromatin *in vivo* in terminally differentiated β cells. Thus, Eed/PRC2 function is required to prevent inappropriate activation of bivalent, accessible genomic regions in terminally differentiated β cells. The data highlight conditional β cell Eed/PRC2 deletion as a model for the transcriptional dysregulation observed in human and mouse diabetes.

Eed/PRC2 Protects Transcription at Epigenomically Unique Lineage Genes

Notably, we observed a select few loci that were *depleted* of H3K27ac marking in KO islets, despite the robust genome-wide deposition (Figure 5E). Consistent with dedifferentiation, these loci included virtually all MβTFs (Pdx1, Nkx6-1, Nkx2-2, Mafa, Pax6, Neurod1, Isl1, and Ucn3; Figure 5E). Interestingly, in our chromatin-state analyses, MβTFs clustered extremely closely within the tSNE projection (Figure S5C), indicating for the first time a common and relatively unique epigenome architecture at these β cell-defining lineage genes: intense and broad active-mark deposition across the entirety of their loci (Figures 1B Nkx6-1 and 5F, 5G, and S5A); lack of silent marks; short gene lengths with few exons; little elongation mark accumulation (H3K36me3/H3K27me1); and, notably, exceptionally broad and uniform Pol2 binding (Figure 1B), indicating distinct transcriptional control. Thus, Eed/PRC2 appears necessary to specifically *protect* transcription at epigenomically unique lineage-defining loci.

Ectopic Transcription Factor Expression Drives Loss of Cell Identity in Min6 Cells

A key signature found in both βEedKO and human T2D islets was the ectopic expression of bivalent chromatin embedded transcription factors normally silenced by PRC2 in healthy islets. To test whether the ectopic activation of these genes was sufficient to drive the loss of cell identity, we identified six factors that were (1) H3K27me3-marked (PRC2-regulated) in both normal mouse and human islets, and (2) markedly upregulated in both

Figure 4. Eed/PRC2 Deficiency Triggers Progressive β Cell Dedifferentiation and Diabetes

(A–C) Blood glucose (top) and insulin (bottom) levels during oral glucose tolerance test (1 g/kg) in βEedKO, control and heterozygote mice from 8 to 25 weeks of age.

(D) Immunofluorescence staining for H3K27me3 (gray), insulin (magenta), and glucagon (green) in βEedKO and control islets. Yellow arrows indicate Ctrl and βEedKO cells harboring or lacking H3K27me3.

(E) Immunofluorescence staining for insulin (magenta) and glucagon (green) on βEedKO islets from 8 to 25 weeks of age.

(F) Representative image of βEedKO islets harboring an RIP-cre-inducible YFP lineage tracer (yellow) stained for insulin (magenta) and glucagon (green). Small white arrow indicates insulin and YFP-positive β cell; large white arrow indicates insulin-negative YFP-positive cell. White dotted line outlines the islet.

(G) Immunofluorescence staining of βEedKO islets for Pdx1, Mafa, Nkx2-2, and Nkx6-1. White dashed lines outline the islets.

(H) Scatterplot of mRNA-seq expression data from βEedKO and control islets. GSEA leading edge gene sets of "Progenitor" (green), "Immature" (magenta), and "Mature" (yellow) pathways are depicted (FDR < 0.05). Embryonic stem cell markers (violet) are not expressed. Dotted lines indicate fragments per kilobase of transcript per million mapped reads = 1.

(I) GSEA analysis of mRNA-seq expression data of all Progenitor (green), Immature (magenta), and Mature (yellow) gene sets in βEedKO versus Ctrl islets. Dotted line indicates FDR-q < 0.05.

(J) Immunofluorescence staining of βEedKO islets for chromogranin-A (green).

(K) Representative immunostaining images for insulin (magenta) and glucagon (green) of βEedKO islets from animals treated with insulin pellet or sham controls for 7 weeks from 18 weeks of age.

(L) Blood glucose levels during oral glucose tolerance test (1 g/kg) of 12-week-old Pdx1-Cre; EedKO (n = 6) or littermate control (n = 5). Each curve represent a single mouse.

(M) Representative image of immunofluorescence staining of mosaic Eed/PRC2 loss-of-function deletion pattern in islets from the normoglycemic (no. 138, lower panel) and hyperglycemic (no. 77, upper panel) Pdx1-Cre; EedKO mice. Arrows indicate nuclei devoid of H3K27me3 signals.

Data represent mean ± SEM. *p < 0.05–0.0001. See also Figure S4.

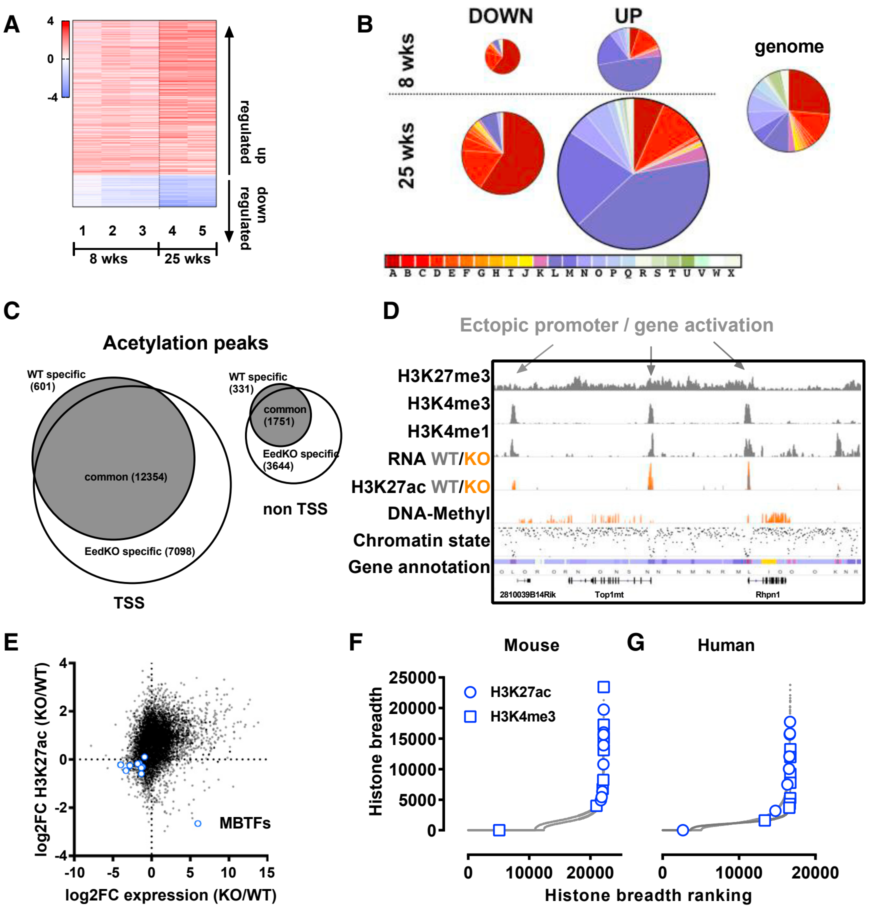

Figure 5. Eed/PRC2 Maintains Global Transcriptional Silencing in Terminally Differentiated β Cells

(A) Clustered heatmap showing log2 fold changes for significantly up- and downregulated genes of βEedKO islet gene expression at both 8 and 25 weeks of age. Samples 1–5 refer to individual βEedKO RNA-seq samples (n = 1–4 per sample).

(B) Pie chart showing chromatin-state distribution of differentially expressed genes at both 8 and 25 weeks of age. Far right panel shows chromatin-state distribution of all genes.

(C) Venn diagram indicating H3K27ac ChIP-seq peaks in βEedKO genome at either TSS (left) or non-TSS- (right) associated genomic loci in wild-type (WT) and βEedKO islets.

(D) Genome browser view of histone mark occupancy and DNA methylation state at accessible promoter and enhancer regions (shaded gray). WT and KO tracks are overlaid for RNA and H3K27ac to facilitate comparison.

(E) Scatterplot of H3K27ac ChIP-seq enrichment signal versus gene expression log2FC of βEedKO versus WT islets. H3K27ac ChIP-seq enrichment signal calculated as area under curve at TSS ± 100 nt (see the STAR Methods). MβTFs are depicted as blue circles.

(F and G) H3K27ac and H3K4me3 ChIP enrichment peak breadth (TSS) rankings in both mouse and human. MβTFs are depicted in blue.

See also Figure S5.

βEedKO and human T2D islets. We co-transfected these six diabetes-specific, Polycomb-regulated ectopic transcription factors (ETFs) (Barx1, Hoxb7, Gata2, Pitx1, Twist1, and Zic1) into the β cell line Min6-B1 and tracked gene expression responses by scRNA-seq, using vectorless and GFP-transfected cells as controls (schematic Figure 6A). We compared 359 single-cell transcriptomes including 50 control, 78 GFP, and 231 ETF overexpressing cells (Figure 6B). Unbiased clustering analysis revealed four clusters. Two were composed mainly of control and GFP-transfected cells showing high insulin and MβTF transcription (Figures 6B and 6C, clusters 1 and 2). The remaining two were comprised of transfected cells exhibiting ETF overexpression and, importantly, loss of MβTF and *Ins1* mRNA expression, and thus, loss of β cell identity (Figures 6B and 6C, clusters 3 and 4). To understand this ETF-driven dedifferentiation, we ordered cells along their dedifferentiation trajectory using pseudotemporal ordering (clusters 1, 2, 3, and 4 in order). We observed two apparent phases of identity loss. First, low ETF expression (as little as 2-fold normal) was coupled to decreasing insulin expression (Figure 6C, region between hashed lines). Importantly, the levels of ectopic ETF expression found able to initiate insulin loss within this 3-day *in vitro* experiment were comparable with those observed in bulk RNA-seq of human diabetic islets, which merges expression profiles of functional and dysfunctional β cells and thus under-represents dysregulation signatures (Figure 6D). In a second phase, more substantial ETF expression tracked with a progressive loss of lineage-defining MβTFs (Figure 6C, right of second dashed line). Also, ectopic transcription factor overexpression triggered downregulation of our epigenomically unique (H3K27ac-broad) class genes, including MβTFs (Figure 6C, bottom panel). Interesting to note, a contrasting hyperactivation of H3K27ac-broad genes was observed during the first phase, indicating once again the high sensitivity of this geneset. These data suggest the existence of multiple distinct transcriptional networks that buffer cellular identity. The model provides a novel system to investigate transcriptional dysregulation relevant to diabetes.

To further validate the *in vivo* relevance of these findings, we searched for ETF overexpression in single-cell RNA-seq data generated from 14-week-old βEedKO β cells (early in the progression toward dedifferentiation and diabetes). Only KO cells (Figure 6E) showed ectopic ETF expression, which, even at moderate levels, was accompanied by loss of insulin and MβTF expression. Thus, ectopic activation of diabetes-specific PRC2-regulated transcription factors is sufficient to drive β cell dedifferentiation.

PRC2-Associated Epigenome Regulation Is Therapeutically Targetable

Finally, we asked to what extent the observed Polycomb-dependent pathology might be amenable to intervention. In a first experiment, we challenged both βEedKO-driven (Figure 7A) and HFD-driven (Figure 7B) pathologies with the histone

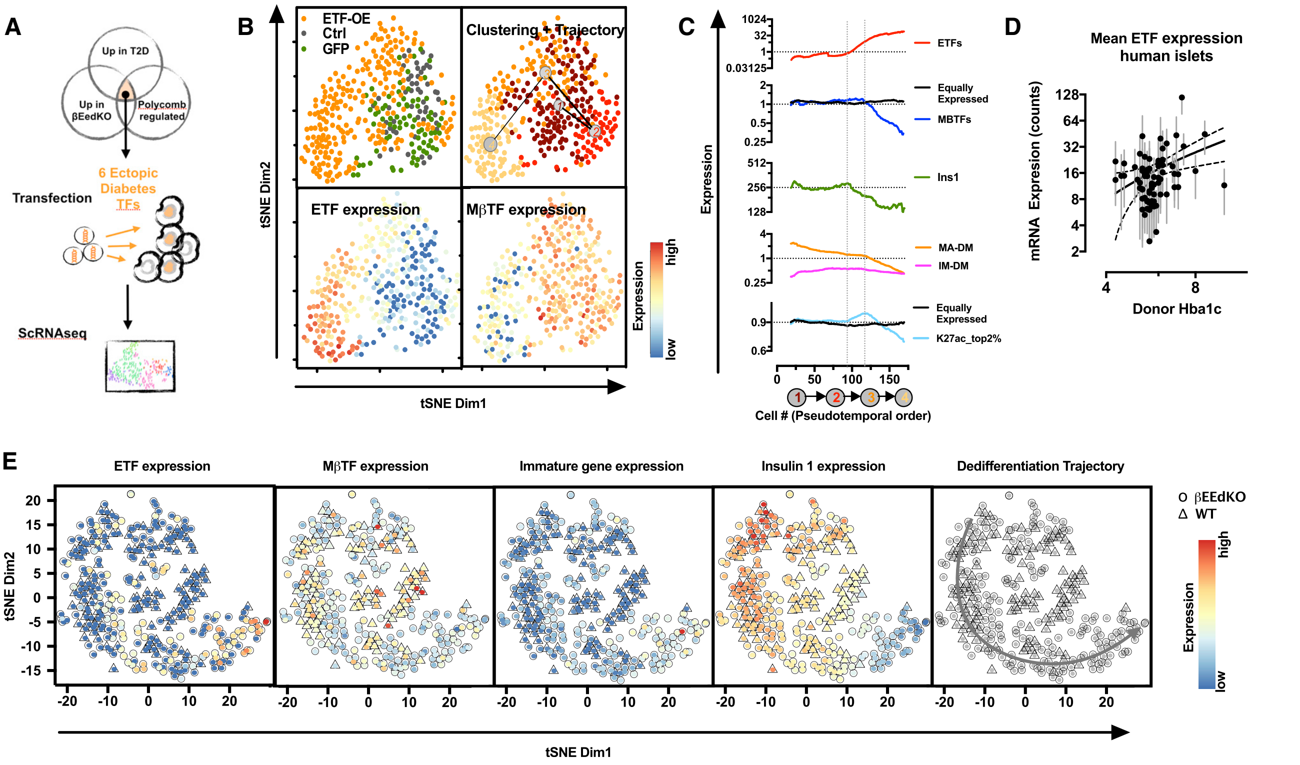

Figure 6. ETF Expression Drives Loss of Cell Identity in Min6 Cells

(A) ETF expression experiment design.

(B) t-SNE representation of control, GFP, and ETF overexpressing cells from scRNA-seq experiment (upper left panel). Four clusters identified by RaceID2 are depicted with a red color gradient (upper right panel). tSNE map superimposed by a color scheme, representing mean ETF expression (lower left panel) and MβTF expression (lower right panel).

(C) Pseudo-temporal ordering of cells along the dedifferentiation trajectory (x axis) and their respective expression levels of ectopic transcription factors (top), MβTFs (second from top), Ins1 (middle); MA-DM, mature gene set from Melton group; IM-DM, immature gene set from Melton group (second from bottom) (Blum et al., 2012), and the top 2% H3K27ac-broad genes (excludes expression outliers Ins1 and Ins2) (bottom).

(D) Mean expression (counts) of ETFs in human islet mRNA sequencing versus Hba1C index. Linear regression (solid line) and 95% confidence interval (dash lines) are shown.

(E) tSNE representation of cells from scRNA-seq experiments on 14-week-old βEedKO (circles) and WT (triangles) islet cells, superimposed by a color scheme, representing mean expression of the depicted gene(sets).

deacetylase inhibitor SAHA. If etiology relies upon ectopic acetylation and transcriptional derepression at sensitive loci, then such an intervention should accelerate dysfunction. Consistent with this idea, SAHA treatment accelerated both HFD- and βEedKO-induced disease (Figures 7A and 7B). These data are consistent with the notion that ectopic acetylation and gene activation contribute to β cell dysfunction *in vivo*. Importantly, they also demonstrate that HFD- and EedKO-driven disease are sensitive to epigenetic interventions.

In a second experiment, we tested whether, by genetically targeting Mll, we might be able to slow the disease progression. Mll is an H3K4 methyltransferase of the COMPASS complex and ortholog of the *Drosophila* Trx gene (Steffen and Ringrose, 2014) originally identified for its ability to genetically oppose Polycomb. If ectopic bivalent gene expression drives pathology, loss of Mll should prevent disease. Using the same Cre system, we generated triple transgenic mice with targeted deletions of Eed, Mll, or both Eed and Mll double KO, in pancreatic β cells. Mll-Eed double KO mice were born healthy and grew normally, indicating intact β cell function. Eed single KO animals (Eed −/−; Mll +/−) developed profound and fully penetrant diabetes, validating the original βEedKO model (Figure 7C). Critically, β cell-specific double KO (Eed −/−; Mll −/−) littermates were largely protected from diabetes, exhibiting improved glucose tolerance and insulin secretion at 25 weeks of age. Importantly, preliminary immunohistochemical analyses revealed substantial protection of the insulin-positive β cell compartment (Figure 7D). These data support the notion that Mll and Eed oppose one another in maintaining β cell identity. Importantly, they demonstrate that the dramatic consequences of PRC2 insufficiency are malleable and theoretically targetable, including by inhibition of Mll.

DISCUSSION

Ten years ago, Yamanaka and colleagues generated induced pluripotent stem cells by overexpressing a handful of transcription factors. The experiment highlighted the complete plasticity potential locked within most differentiated cells and highlighted

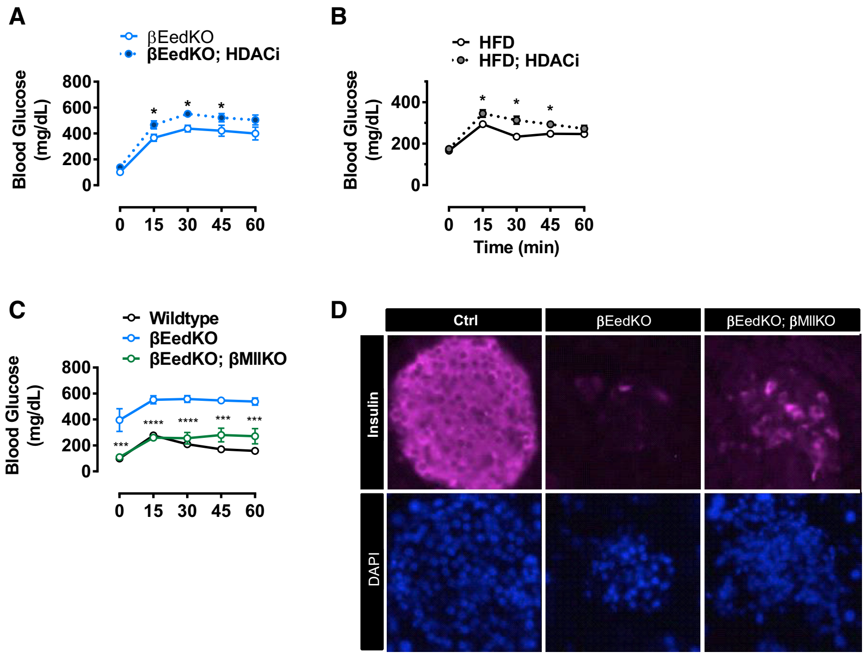

Figure 7. PRC2-Associated Epigenome Regulation Is Therapeutically Targetable

(A) Blood glucose levels during oral glucose tolerance test (1 g/kg) of 25-week-old βEedKO mice treated orally for 8 weeks with SAHA (2 mg/day) or vehicle control (n = 4–6 mice per group).

(B) Blood glucose levels during oral glucose tolerance test (1 g/kg) of 24-week-old HFD-mice treated orally for 8 weeks with SAHA (2 mg/day) or vehicle control (n = 10 mice per group).

(C) Blood glucose levels during oral glucose tolerance test (1 g/kg) of 25- to 30-week-old control, βEedKO, and βEedKO; βMllKO double knockout mice (DKO). Animals were sex-matched littermates (n = 3–4 mice per group). Statistical analysis indicated refers to βEedKO and βEedKO; βMllKO DKO mice comparisons.

(D) Representative immunofluorescence images of Ctrl, βEedKO, and βEedKO; βMllKO DKO mice islets stained for insulin and DAPI at 25 weeks of age.

Data are mean ± SEM. *p < 0.05, ***p < 0.001, ****p < 0.0001.

the importance of mechanisms that maintain terminal cell identity. Indeed, the closer cells are to terminal differentiation, the more reinforced such mechanisms appear to be (Pasque et al., 2011). A number of studies have highlighted the importance of epigenetic regulation in active maintenance of β cell differentiation (Gao et al., 2014; Talchai et al., 2012; Dhawan et al., 2011). Here we add the Polycomb-group protein Eed, and thus the PRC2 complex, to that list highlighting a novel role for PRC2 in maintaining functional β cell identity. We observed chromatin-state-associated transcriptional dysregulation in mouse and human T2D characterized by inappropriate and broad activation of progenitor and immature β cell programs, both consistent with the notion of β cell dedifferentiation and loss of β cell function. Importantly, *Eed* KO, which abrogates PRC2 enzymatic and reader capacities, resulted in transcriptional and pathophysiological changes that recapitulate human and mouse β cell dysfunction and diabetes, namely dysregulation of bivalent and chromatin state A embedded genes, and progressive loss of terminal β cell identity. The data highlight the importance of PRC2 for maintaining proper gene expression in both poised and active regions, and indicate that fundamental transcriptional dysregulation is a novel driving feature of β cell dysfunction.

The finding that PRC2 loss triggers diabetes-mimicking transcriptional dysregulation and dedifferentiation warrants deeper investigations into relative *Ezh1-, Ezh2-* and non-methyltransferase-dependent Polycomb activities in the β cell compartment. They also beg exploration of more subtle Polycomb-deficient models such as βEedKO-heterozygotes and adult-inducible deficiencies, deletions of alternate complex members, as well as dissection of the competing Mll-containing complexes. Combining these approaches will ultimately define the relative importance of H3K27me2/3 loss, ectopic gene activation, and cell turnover toward maintenance of optimal terminal differentiation.

The chromatin-state analysis as presented (Figure 1F) highlights underappreciated epigenomic diversity in transcriptional control at both active and silent genes. Interesting for β cell biologists, a unique epigenome architecture characterized by extremely broad active marking was observed at virtually all MβTF lineage factors. Interestingly, our data identify these loci as among the most sensitive to transcriptional collapse during human and mouse β cell dysfunction and dedifferentiation. The late *down*regulation and relative loss of active mark enrichment of these loci in *Eed* KO mice suggest that PRC2 also acts to ensure "transcriptional focusing" at these unique loci. Recent work indicates that spatial confinement is the key parameter controlling the frequency of genomic encounters and building of locus-control regions (superenhancers) and transcription factories (Levine, 2014; Benayoun et al., 2014). Our data are consistent with the idea that H3K27me2/3, which decorates nearly 80% of the genome, enhances 3D compartmentalization of transcriptional regulatory machinery (Boettiger et al., 2016; Ferrari et al., 2014), and that the highly active subset of chromatin state A embedded genes are particularly dependent on such regulation. Our data add to a select few examples in disparate cell systems where PRC2 actually promotes transcription (Di Croce and Helin, 2013). In addition to stabilizing β cell progenitor gene silencing upon maturation, PRC2 therefore appears to enhance terminal differentiation by ensuring transcriptional fidelity of chromatin state A embedded lineage-defining regulators.

Previous reports have indicated glucotoxicity as an important trigger for dedifferentiation (Wang et al., 2014; Guo et al., 2013; Bensellam et al., 2017). Our findings from insulin-pelleted βEedKO animals, as well as from our mosaic/normoglycemic Pdx-EedKO mice, demonstrate that PRC2-associated dedifferentiation is cell autonomous and does not require hyperglycemia. The data demonstrate that Eed deficiency alone is sufficient to drive terminal dedifferentiation. It will be important now to investigate potential synergistic interactions between PRC2

insufficiency and hyperglycmia in diabetes. Indeed, we consider a two-hit model for dedifferentiation, one in which hyperglycemia and PRC2 insufficiency compound one another to drive dedifferentiation, as most likely.

In summary, the data presented here highlight chromatin-state-specific transcriptional dysregulation, dedifferentiation, and alternate lineage activation as key transcriptional features of β cell dysfunction. They demonstrate that Polycomb-mediated gene silencing maintains the specificity of the β cell program by both repression of immature/alternate lineage potential and focusing of transcription at key MβTFs. The findings indicate that PRC2 functions chronically in β cells, that PRC2-insufficiency contributes to the pathology of human T2D, and, therefore, that inhibition of PRC2-opposing factors (e.g., *Mll*, *Jmjd3*, and *Utx*) might serve as potential therapeutic strategy for T2D.

Limitation of the Study

We show here that Eed/PRC2 plays a critical role in safe-guarding functional β cell identity. Since, practically, it is challenging to assemble analyses on large numbers of fresh or fixed human islet preparations that are highly homogeneous in their postmortem conditions, we chose to measure H3K27me3 levels in fixed pancreatic samples graciously provided by nPOD (Figures 2I–2K). It will be important to rule out any postmortem influences on the conclusions here, using *in vivo* reporters in mice or samples derived from surgery in humans. Also, for practical reasons, we used an overexpression system to test ETF involvement in β cell de-differentiation. In humans this process is presumably much more subtle, combines with dysglycemia, and has many years to occur. Indeed, the relative timing and contribution in human diabetes of impaired chromatin silencing, ETF expression and hyperglycemia cannot be concluded from our study. These represent important goals for future study.

STAR★METHODS

Detailed methods are provided in the online version of this paper and include the following:

- KEY RESOURCES TABLE
- CONTACT FOR REAGENT AND RESOURCE SHARING
- EXPERIMENTAL MODEL AND SUBJECT DETAILS
  - βEedKO, βEzh2KO, βEedMllDKO Pdx1-EedKO and Lineage-Tracing βEedKO-YFP Mouse Model
  - Cell Line Model
  - Human Pancreatic Histological Samples
- METHOD DETAILS
  - Animal Husbandry
  - Mouse Embryo Isolation, Section Preparation and Staging
  - Glucose Tolerance Test and Plasma Insulin Measurements
  - Islet Isolation
  - Histological Preparation of the Pancreas
  - Immunofluorescence Stainings and Image Acquisition
  - Total RNA Extraction
  - Bulk RNA-seq and ChIP-seq
  - Transcription Factor Over-Expression
  - Single Cell RNA-seq
  - HDACi Administration
- QUANTIFICATION AND STATISTICAL ANALYSIS
  - Bulk RNA-seq Bioinformatic Analysis
  - Single Cell RNA-seq Analysis
  - Gene-Set Enrichment Analysis
  - ChIP-Sequencing Bioinformatics Analysis
  - Chromatin Segmentation
  - Image Quantifications
  - Statistical Analysis
- DATA AND SOFTWARE AVAILABILITY

SUPPLEMENTAL INFORMATION

Supplemental Information includes five figures and five tables and can be found with this article online at https://doi.org/10.1016/j.cmet.2018.04.013.

ACKNOWLEDGMENTS

The authors thank S. Kubicek, G. Gradwohl, R. Schneider, T. Jenuwein, A. Öst, K. Gossens, J. Longinotto, and S. Welz for discussion, technical and analytical help, and advice. This research was performed with the support of the Network for Pancreatic Organ Donors with Diabetes (nPOD), a collaborative type 1 diabetes research project sponsored by the Juvenile Diabetes Research Foundation International (JDRF). Organ Procurement Organizations partnering with nPOD to provide research resources are listed at www.jdrfnpod.org/our-partners.php. This work was supported by funding from the Max Planck Society, ERC (ERC-StG-281641; ERC-CoG-682679), BMBF (DEEP; EpiTriO 01KU1501A), MINECO (BFU2015_70581), AGAUR (2015FI_B_00632), and EU_FP7 (NoE "Epigenesys").

AUTHOR CONTRIBUTIONS

Concept, T.T.-H.L. and J.A.P.; Methodology, T.T.-H.L., S., J.A.P., H.X., and S.H.O.; Software, S.H., E.C., and D.G.; Formal Analysis, Investigation, and Visualization, T.T.-H.L., S.H., E.D., T.B., S., L.L., J.A.P., E.C., C.-H.Y., L.E., K.D., L.A., M.S., U.B., R.T., M.R., S.J.R., F.C.L., B.G.H., and A.M.L.; Writing – Original Draft, T.T.-H.L. and J.A.P.; Writing – Review & Editing, T.T.-H.L., J.A.P., A.M.L., and F.C.L.; Supervision, Resources, and Funding Acquisition, J.A.P., T.V., and D.G.

DECLARATION OF INTERESTS

The authors declare no competing interests.

Received: October 13, 2017
Revised: January 10, 2018
Accepted: April 12, 2018
Published: May 10, 2018

REFERENCES

Arda, H.E., Benitez, C.M., and Kim, S.K. (2013). Gene regulatory networks governing pancreas development. Dev. Cell *25*, 5–13.

Arrigoni, L., Richter, A.S., Betancourt, E., Bruder, K., Diehl, S., Manke, T., and Bönisch, U. (2015). Standardizing chromatin research: a simple and universal method for ChIP-seq. Nucleic Acids Res. *44*, e67.

Avrahami, D., Li, C., Zhang, J., Schug, J., Avrahami, R., Rao, S., Stadler, M.B., Burger, L., Schübeler, D., Glaser, B., et al. (2015). Aging-dependent demethylation of regulatory elements correlates with chromatin state and improved β cell function. Cell Metab. *22*, 619–632.

Benayoun, B.A., Pollina, E.A., Ucar, D., Mahmoudi, S., Karra, K., Wong, E.D., Devarajan, K., Daugherty, A.C., Kundaje, A.B., Mancini, E., et al. (2014). H3K4me3 breadth is linked to cell identity and transcriptional consistency. Cell *158*, 673–688.

Benito, E., Urbanke, H., Ramachandran, B., Barth, J., Halder, R., Awasthi, A., Jain, G., Capece, V., Burkhardt, S., Navarro-Sala, M., et al. (2015). HDAC

inhibitor-dependent transcriptome and memory reinstatement in cognitive decline models. J. Clin. Invest. *125*, 3572–3584.

Bensellam, M., Jonas, J.-C., and Laybutt, D.R. (2017). Mechanisms of β-cell dedifferentiation in diabetes: recent findings and future research directions. J. Endocrinol. *236*, R109–R143.

Blum, B., Hrvatin, S.S.Š., Schuetz, C., Bonal, C., Rezania, A., and Melton, D.A. (2012). Functional beta-cell maturation is marked by an increased glucose threshold and by expression of urocortin 3. Nat. Biotechnol. *30*, 261–264.

Boettiger, A.N., Bintu, B., Moffitt, J.R., Wang, S., Beliveau, B.J., Fudenberg, G., Imakaev, M., Mirny, L.A., Wu, C.-T., and Zhuang, X. (2016). Super-resolution imaging reveals distinct chromatin folding for different epigenetic states. Nature *529*, 418–422.

Bramswig, N.C., Everett, L.J., Schug, J., Dorrell, C., Liu, C., Luo, Y., Streeter, P.R., Naji, A., Grompe, M., and Kaestner, K.H. (2013). Epigenomic plasticity enables human pancreatic α to β cell reprogramming. J. Clin. Invest. *123*, 1275–1284.

Brereton, M.F., Iberl, M., Shimomura, K., Zhang, Q., Adriaenssens, A.E., Proks, P., Spiliotis, I.I., Dace, W., Mattis, K.K., Ramracheya, R., et al. (2014). Reversible changes in pancreatic islet structure and function produced by elevated blood glucose. Nat. Commun. *5*, 4639.

Chen, H., Gu, X., Su, I.-H., Bottino, R., Contreras, J.L., Tarakhovsky, A., and Kim, S.K. (2009). Polycomb protein Ezh2 regulates pancreatic beta-cell Ink4a/Arf expression and regeneration in diabetes mellitus. Genes Dev. *23*, 975–985.

Cinti, F., Bouchi, R., Kim-Muller, J.Y., Ohmura, Y., Sandoval, P.R., Masini, M., Marselli, L., Suleiman, M., Ratner, L.E., Marchetti, P., et al. (2016). Evidence of β-cell dedifferentiation in human type 2 diabetes. J. Clin. Endocrinol. Metab. *101*, 1044–1054.

Cnop, M., Igoillo Esteve, M., Hughes, S.J., Walker, J.N., Cnop, I., and Clark, A. (2011). Longevity of human islet α- and β-cells. Diabetes Obes. Metab. *13*, 39–46.

Collombat, P., Xu, X., Ravassard, P., Sosa-Pineda, B., Dussaud, S., Billestrup, N., Madsen, O.D., Serup, P., Heimberg, H., and Mansouri, A. (2009). The ectopic expression of Pax4 in the mouse pancreas converts progenitor cells into alpha and subsequently beta cells. Cell *138*, 449–462.

Di Croce, L., and Helin, K. (2013). Transcriptional regulation by Polycomb group proteins. Nat. Struct. Mol. Biol. *20*, 1147–1155.

Dhawan, S., Georgia, S., Tschen, S.-I., Fan, G., and Bhushan, A. (2011). Pancreatic β cell identity is maintained by DNA methylation-mediated repression of Arx. Dev. Cell *20*, 419–429.

Ediger, B.N., Lim, H.-W., Juliana, C., Groff, D.N., Williams, L.T., Dominguez, G., Liu, J.-H., Taylor, B.L., Walp, E.R., Kameswaran, V., et al. (2017). LIM domain-binding 1 maintains the terminally differentiated state of pancreatic β cells. J. Clin. Invest. *127*, 215–229.

Ezhkova, E., Lien, W.-H., Stokes, N., Pasolli, H.A., Silva, J.M., and Fuchs, E. (2011). EZH1 and EZH2 cogovern histone H3K27 trimethylation and are essential for hair follicle homeostasis and wound repair. Genes Dev. *25*, 485–498.

Fadista, J., Vikman, P., Laakso, E.O., Mollet, I.G., Esguerra, J.L., Taneera, J., Storm, P., Osmark, P., Ladenvall, C., Prasad, R.B., et al. (2014). Global genomic and transcriptomic analysis of human pancreatic islets reveals novel genes influencing glucose metabolism. Proc. Natl. Acad. Sci. USA *111*, 13924–13929.

Ferrari, K.J., Scelfo, A., Jammula, S., Cuomo, A., Barozzi, I., Stützer, A., Fischle, W., Bonaldi, T., and Pasini, D. (2014). Polycomb-dependent H3K27me1 and H3K27me2 regulate active transcription and enhancer fidelity. Mol. Cell *53*, 49–62.

Gao, T., McKenna, B., Li, C., Reichert, M., Nguyen, J., Singh, T., Yang, C., Pannikar, A., Doliba, N., Zhang, T., et al. (2014). Pdx1 maintains β cell identity and function by repressing an α cell program. Cell Metab. *19*, 259–271.

Grün, D., Muraro, M.J., Boisset, J.-C., Wiebrands, K., Lyubimova, A., Dharmadhikari, G., van den Born, M., van Es, J., Jansen, E., Clevers, H., et al. (2016). De novo prediction of stem cell identity using single-cell transcriptome data. Cell Stem Cell *19*, 266–277.

Guo, S., Dai, C., Guo, M., Taylor, B., Harmon, J.S., Sander, M., Robertson, R.P., Powers, A.C., and Stein, R. (2013). Inactivation of specific β cell transcription factors in type 2 diabetes. J. Clin. Invest. *123*, 3305–3316.

Gutiérrez, G.D., Bender, A.S., Cirulli, V., Mastracci, T.L., Kelly, S.M., Tsirigos, A., Kaestner, K.H., and Sussel, L. (2017). Pancreatic β cell identity requires continual repression of non-β cell programs. J. Clin. Invest. *127*, 244–259.

Hashimshony, T., Senderovich, N., Avital, G., Klochendler, A., de Leeuw, Y., Anavy, L., Gennert, D., Li, S., Livak, K.J., Rozenblatt-Rosen, O., et al. (2016). CEL-Seq2: sensitive highly-multiplexed single-cell RNA-seq. Genome Biol. *17*, 77.

Hoffman, B.G., Robertson, G., Zavaglia, B., Beach, M., Cullum, R., Lee, S., Soukhatcheva, G., Li, L., Wederell, E.D., Thiessen, N., et al. (2010). Locus co-occupancy, nucleosome positioning, and H3K4me1 regulate the functionality of FOXA2-, HNF4A-, and PDX1-bound loci in islets and liver. Genome Res. *20*, 1037–1051.

Holmberg, J., and Perlmann, T. (2012). Maintaining differentiated cellular identity. Nat. Rev. Genet. *13*, 429–439.

Johnston, N.R., Mitchell, R.K., Haythorne, E., Pessoa, M.P., Semplici, F., Ferrer, J., Piemonti, L., Marchetti, P., Bugliani, M., Bosco, D., et al. (2016). Beta cell hubs dictate pancreatic islet responses to glucose. Cell Metab. *24*, 389–401.

Landsman, L., Parent, A., and Hebrok, M. (2011). Elevated Hedgehog/Gli signaling causes beta-cell dedifferentiation in mice. Proc. Natl. Acad. Sci. USA *108*, 17010–17015.

Laybutt, D.R., Glandt, M., Xu, G., Ahn, Y.B., Trivedi, N., Bonner-Weir, S., and Weir, G.C. (2003). Critical reduction in beta-cell mass results in two distinct outcomes over time. Adaptation with impaired glucose tolerance or decompensated diabetes. J. Bio. Chem. *278*, 2997–3005.

Levine, M. (2014). The contraction of time and space in remote chromosomal interactions. Cell *158*, 243–244.

Mammana, A., and Chung, H.-R. (2015). Chromatin segmentation based on a probabilistic model for read counts explains a large portion of the epigenome. Genome Biol. *16*, 436.

Margueron, R., and Reinberg, D. (2011). The Polycomb complex PRC2 and its mark in life. Nature *469*, 343–349.

Oliver-Krasinski, J.M., and Stoffers, D.A. (2008). On the origin of the beta cell. Genes Dev. *22*, 1998–2021.

Papizan, J.B., Singer, R.A., Tschen, S.-I., Dhawan, S., Friel, J.M., Hipkens, S.B., Magnuson, M.A., Bhushan, A., and Sussel, L. (2011). Nkx2.2 repressor complex regulates islet β-cell specification and prevents β-to-α-cell reprogramming. Genes Dev. *25*, 2291–2305.

Pasque, V., Jullien, J., Miyamoto, K., Halley-Stott, R.P., and Gurdon, J.B. (2011). Epigenetic factors influencing resistance to nuclear reprogramming. Trends Genet. *27*, 516–525.

Puri, S., Akiyama, H., and Hebrok, M. (2013). VHL-mediated disruption of Sox9 activity compromises β-cell identity and results in diabetes mellitus. Genes Dev. *27*, 2563–2575.

Ramírez, F., Ryan, D.P., Grüning, B., Bhardwaj, V., Kilpert, F., Richter, A.S., Heyne, S., Dündar, F., and Manke, T. (2016). deepTools2: a next generation web server for deep-sequencing data analysis. Nucleic Acids Res. *44*, W160–W165.

Russ, H.A., Bar, Y., Ravassard, P., and Efrat, S. (2008). In vitro proliferation of cells derived from adult human beta-cells revealed by cell-lineage tracing. Diabetes *57*, 1575–1583.

Sabatini, P.V., Speckmann, T., Nian, C., Glavas, M.M., Wong, C.K., Yoon, J.S., Kin, T., Shapiro, A.M.J., Gibson, W.T., Verchere, C.B., et al. (2018). Neuronal PAS domain protein 4 suppression of oxygen sensing optimizes metabolism during excitation of neuroendocrine cells. Cell Rep. *22*, 163–174.

Schuettengruber, B., and Cavalli, G. (2009). Recruitment of Polycomb group complexes and their role in the dynamic regulation of cell fate choice. Development *136*, 3531–3542.

Simon, J.A., and Kingston, R.E. (2013). Occupying chromatin: polycomb mechanisms for getting to genomic targets, stopping transcriptional traffic, and staying put. Mol. Cell *49*, 808–824.

Stanger, B.Z., and Hebrok, M. (2013). Control of cell identity in pancreas development and regeneration. Gastroenterology *144*, 1170–1179.

Steffen, P.A., and Ringrose, L. (2014). What are memories made of? How polycomb and trithorax proteins mediate epigenetic memory. Nat. Rev. Mol. Cell Biol. *15*, 340–356.

Swisa, A., Avrahami, D., Eden, N., Zhang, J., Feleke, E., Dahan, T., Cohen-Tayar, Y., Stolovich-Rain, M., Kaestner, K.H., Glaser, B., et al. (2017). PAX6 maintains β cell identity by repressing genes of alternative islet cell types. J. Clin. Invest. *127*, 230–243.

Szabat, M., Lynn, F.C., Hoffman, B.G., Kieffer, T.J., Allan, D.W., and Johnson, J.D. (2012). Maintenance of β-cell maturity and plasticity in the adult pancreas: developmental biology concepts in adult physiology. Diabetes *61*, 1365–1371.

Talchai, C., Xuan, S., Lin, H.V., Sussel, L., and Accili, D. (2012). Pancreatic β cell dedifferentiation as a mechanism of diabetic β cell failure. Cell *150*, 1223–1234.

Taylor, B.L., Liu, F.-F., and Sander, M. (2013). Nkx6.1 is essential for maintaining the functional state of pancreatic beta cells. Cell Rep. *4*, 1262–1275.

Tennant, B.R., Robertson, A.G., Kramer, M., Li, L., Zhang, X., Beach, M., Thiessen, N., Chiu, R., Mungall, K., Whiting, C.J., et al. (2013). Identification and analysis of murine pancreatic islet enhancers. Diabetologia *56*, 542–552.

van Arensbergen, J., García-Hurtado, J., Moran, I., Maestro, M.A., Xu, X., Van de Casteele, M., Skoudy, A.L., Palassini, M., Heimberg, H., and Ferrer, J. (2010). Derepression of Polycomb targets during pancreatic organogenesis allows insulin-producing beta-cells to adopt a neural gene activity program. Genome Res. *20*, 722–732.

van Arensbergen, J., García-Hurtado, J., Maestro, M.A., Correa-Tapia, M., Rutter, G.A., Vidal, M., and Ferrer, J. (2013). Ring1b bookmarks genes in pancreatic embryonic progenitors for repression in adult β cells. Genes Dev. *27*, 52–63.

Wang, Z., York, N.W., Nichols, C.G., and Remedi, M.S. (2014). Pancreatic β cell dedifferentiation in diabetes and redifferentiation following insulin therapy. Cell Metab. *19*, 872–882.

Weir, G.C., Aguayo-Mazzucato, C., and Bonner-Weir, S. (2013). β-Cell dedifferentiation in diabetes is important, but what is it? Islets *5*, 233–237.

World Health Organization (WHO) (2016). Global Report on Diabetes (WHO).

Xie, R., Everett, L.J., Lim, H.-W., Patel, N.A., Schug, J., Kroon, E., Kelly, O.G., Wang, A., D'Amour, K.A., Robins, A.J., et al. (2013). Dynamic chromatin remodeling mediated by polycomb proteins orchestrates pancreatic differentiation of human embryonic stem cells. Cell Stem Cell *12*, 224–237.

Xie, H., Xu, J., Hsu, J.H., Nguyen, M., Fujiwara, Y., Peng, C., and Orkin, S.H. (2014). Polycomb repressive complex 2 regulates normal hematopoietic stem cell function in a developmental-stage-specific manner. Cell Stem Cell *14*, 68–80.

Xu, C.-R., Cole, P.A., Meyers, D.J., Kormish, J., Dent, S., and Zaret, K.S. (2011). Chromatin "prepattern" and histone modifiers in a fate choice for liver and pancreas. Science *332*, 963–966.

Ziv, O., Glaser, B., and Dor, Y. (2013). The plastic pancreas. Dev. Cell *26*, 3–7.

STAR★METHODS

KEY RESOURCES TABLE

REAGENT or RESOURCE	SOURCE	IDENTIFIER
Antibodies		
α-H3K27me3 (ChIP-seq)	Diagenode	Cat# C15410195
α-H3K27me3 (Immunofluorescence)	Gift from Dr. T. Jenuwein	N/A
α-H3K9me3	Diagenode	Cat# C15410193
α-H3K27ac	Diagenode	Cat# C15410196; RRID:AB_2637079
α-H3K4me3	Diagenode	Cat #pAb-003-050; RRID:AB_2616052
α-H3K36me3	Diagenode	Cat #pAb-192-050
α-H2AK119ub	Cell Signaling	Cat# 8240; RRID:AB_10891618
α-H3K27me2	Diagenode	Cat# C15410046
α-H3K27me1	Diagenode	Cat# C15410045
α-H3	Active Motif	Cat# 39763; RRID:AB_2650522
α-Pol-II	Diagenode	Cat #C15200004
α-Insulin	DAKO	Cat# A056401-2; AB_2617169
α-Glucagon	Novus	Cat# K79bB10; AB_790003
α-Pancreatic Polypeptide	Novus	Cat# NB100-1793; RRID:AB_2268669
α-Somatostatin	Bio-Rad/AbD Serotec	Cat# 8330-0009; RRID:AB_2195908
α-Chromogranin A	Thermo Fisher Scientific	Cat# PA1-37445; RRID:AB_2080987
α-Nkx6-1	Dev. Studies Hybridoma Bank	Cat# F55A12; RRID:AB_532379
α-MafA	Bethyl Laboratories	Cat# IHC-00352; RRID:AB_1279486
α-Nkx2-2	Dev. Studies Hybridoma Bank	Cat# 74.5A5; RRID:AB_531794
α-Pdx1	Dev. Studies Hybridoma Bank	Cat# F6A11; RRID:AB_1157904
α-GFP/YFP	Thermo Fisher Scientific	Cat#A-11122; RRID:AB_221569
Biological Samples		
Human pancreas donor paraffin blocks	Network for Pancreatic Organ Donors with Diabetes	http://www.jdrfnpod.org/; RRID: SCR_014641
Chemicals, Peptides, and Recombinant Proteins		
SAHA, MK0683	Selleckchem	Cat#S1047
β-Cyclodextrin	Sigma	Cat#332607
TRI Reagent	Sigma	Cat#T9424
Glyco Blue Coprecipitant	Thermo Fisher Scientific	Cat#AM9516
Vapor-Lock	Qiagen	Cat#981611
AMPure XP beads	Beckman Coulter	Cat#A63881
Agencount RNAClean beads	Beckman Coulter	Cat#A63987
Roti-HistoFix 4%	Carl Roth	Cat#P087.1
HistoVT One	Nacalai Tesque	Cat#06380-05
RPMI-1640 Medium with GlutMAX supplement	Thermo Fisher Scientific	Cat#61870-010
Collegenase XI	Sigma	Cat #C7657
Histopaque (1.077 g/mL)	Sigma	Cat# 10771
Critical Commercial Assays		
Mercodia Ultrasensitive Insulin ELISA	Mercodia	Cat#10-1132-01
MEGAscript T7 Transcription Kit	Thermo Fisher Scientific	Cat# AM1334
Illumina Tru Seq RNA Sample Prep Kit v2	Illumina	Cat#RS-122-2001
Amaxa Cell Line Nucleofector Kit T	Lonza	Cat#VCA-1002
DeadEnd Fluorometric TUNEL System	Promega	Cat#G3250

(*Continued on next page*)

Continued

REAGENT or RESOURCE	SOURCE	IDENTIFIER
Deposited Data		
H3K27ac, H3K27me3, H3K4me3, H3K9me3, H3K27me1, H3K36me3, H3K27me2, H2AK119Ub, Pol-II ChIP-seq data of mouse pancreatic islets (Details in Table S4)	This manuscript	GEO: GSE110648
H3K4me1, H3K9me3, H3K27me3 ChIP-seq data of WT mouse pancraetic islets (Details in Table S4)	Hoffman et al., 2010; Tennant et al., 2013	NCBI: SRA008281; GEO: GSE30298
RNA-seq data of Ctrl and βEedKO mouse islets	This manuscript	GEO: GSE110648
Single cell RNA-seq data of chow and HFD treated mouse islets	This manuscript	GEO: GSE110648
Single cell RNA-seq data of βEedKO mouse islets	This manuscript	GEO: GSE110648
Single cell RNA-seq data of Min6-B1 cell line	This manuscript	GEO: GSE110648
DNA-methylation (WGSBS) data of mouse β cells	Avrahami et al., 2015	GEO: GSE68618
Human β cell RNA-seq data	Fadista et al., 2014	GEO: GSE50398
Human β cell H3K4me3, H3K27me3 ChIP-seq data	Bramswig et al., 2013	GEO: GSE50386
Experimental Models: Cell Lines		
Min6-B1	Gift from Dr. Philip Halban	N/A
Experimental Models: Organisms/Strains		
RIP-cre line (B6.Cg-Tg(Ins2-cre)25Mgn/J)	Gift from Dr. Pedro Herrera	Jax strain #003573; RRID:IMSR_JAX:003573
PDX-1-cre line (STOCK Tg(Pdx1-cre/Esr1*)#Dam/J)	Gift from Dr. Gerald Gradwohl	Jax strain #024968; RRID:IMSR_JAX:024968
Eed$^{flox/flox}$ line (B6;129S1-Eed^{tm1Sho}/J)	Gift from Dr. Stuart Orkin	Jax strain #022727; RRID:IMSR_JAX:022727
Ezh2$^{flox/flox}$ line (STOCK Ezh2^{tm2Sho}/J)	Gift from Dr. Stuart Orkin	Jax strain #022616; RRID:IMSR_JAX:022616
Mll$^{flox/flox}$ line (Kmt2atm1.1Erns)	Gift from Dr. Patricia Ernst	N/A
YFP-reporter line (B6.129X1-Gt(ROSA) 26So$^{rtm1(EYFP)Cos}$/J)	Gift from Dr. Thomas Boehm	Jax strain #006148; RRID:IMSR_JAX:006148
Oligonucleotides		
Cel-seq2 oligodT: GCCGGTAATACGACTC ACTATAGGGAGTTCTACAGTCC GACGA TCNNNNNN[6 base barcode]TTTTTTT TTTTTTTTTTTTTTTTTV	Hashimshony et al., 2016	N/A
Cel-seq2 RT library primer: GCCTTGGCA CCCGAGAATTCCANNNNNN	Hashimshony et al., 2016	N/A
Recombinant DNA		
Hoxb7 (NM_010460) Mouse Tagged ORF Clone	Origene	MR223869
Zic1 (NM_009573) Mouse Tagged ORF Clone	Origene	MR207138
Barx1 (NM_007526) Mouse Tagged ORF Clone	Origene	MR222169
Gata2 (NM_008090) Mouse Tagged ORF Clone	Origene	MR226728
Pitx1 (NM_011097) Mouse Tagged ORF Clone	Origene	MR204502
Twist1 (NM_011658) Mouse Tagged ORF Clone	Origene	MR227370
Software and Algorithms		
Prism 7	Graphpad Software	http://www.graphpad.com; RRID: SCR_002798
ImageJ	Image J	https://imagej.nih.gov/ij/; RRID:SCR_003070
CellProfiler Image Analysis software	Broad Institute	http://cellprofiler.org/;RRID:SCR_003070
GSEA	Broad Institute	http://software.broadinstitute.org/gsea; RRID:SCR_003199

(*Continued on next page*)

Continued

REAGENT or RESOURCE	SOURCE	IDENTIFIER
Bowtie2 (v2.2.8)	https://doi.org/10.1038/nmeth.1923	http://bowtie-bio.sourceforge.net/bowtie2/index.shtml; RRID:SCR_005476
DeepTools (v2.4.1)	https://doi.org/10.1093/nar/gkw257	https://github.com/deeptools/deepTools
MACS2 (v2.1.1.20160309)	https://doi.org/10.1186/gb-2008-9-9-r137	https://github.com/taoliu/MACS; RRID:SCR_013291
TopHat (v2.0.13)	https://doi.org/10.1186/gb-2013-14-4-r36	http://tophat.cbcb.umd.edu/; RRID:SCR_013035
featureCounts (subread-1.5.0-p1)	https://doi.org/10.1093/bioinformatics/btt656	http://bioinf.wehi.edu.au/featureCounts; RRID:SCR_012919
edgeR (v3.14)	https://doi.org/10.1093/bioinformatics/btp616	http://www.bioconductor.org/packages/release/bioc/html/edgeR.html; RRID:SCR_012802
Isolator	https://doi.org/10.1101/088765	https://github.com/dcjones/isolator
DESeq2 (v1.8.1)	http://doi.org/10.1186/s13059-014-0550-8	http://bioconductor.org/packages/release/bioc/html/DESeq2.html; RRID:SCR_000154
Cutadapt (v1.9.1)	https://doi.org/10.14806/ej.17.1.200	https://cutadapt.readthedocs.io/en/stable/; RRID:SCR_011841
STAR (v2.5.3.a)	https://doi.org/10.1093/bioinformatics/bts635	https://github.com/alexdobin/STAR; RRID:SCR_015899
RaceID2/StemID2	https://doi.org/10.1038/nature14966	https://github.com/dgrun/RaceID
EpicSeg	Mammana and Chung, 2015 https://doi.org/10.1186/s13059-015-0708-z	https://github.com/lamortenera/epicseg
MethylDackel	MethylDackel	https://github.com/dpryan79/MethylDackel

CONTACT FOR REAGENT AND RESOURCE SHARING

Further information and requests for resources and reagents should be directed to and will be fulfilled by the Lead Contact, J. Andrew Pospisilik (pospisilik@ie-freiburg.mpg.de).

EXPERIMENTAL MODEL AND SUBJECT DETAILS

βEedKO, βEzh2KO, βEedMllDKO Pdx1-EedKO and Lineage-Tracing βEedKO-YFP Mouse Model

Breeding pairs of RIP-cre (Tg(Ins2-cre)23Herr), PDX-1-cre, *Eed*$^{flox/flox}$, Ezh2$^{flox/flox}$, Mll$^{flox/flox}$and YFP-reporter (B6.129X1-Gt(ROSA) 26Sortm1(EYFP)Cos/J) transgenic mouse line (C57B6/J) were kindly provided by Pedro Herrera, Gerald Gradwohl, Stuart Orkin, Petricia Ernst, and Thomas Boehm, respectively. To generate βEedKO animals, *Eed*$^{flox/flox}$ animals were crossed with RIP-cre positive *Eed*$^{+/flox}$ animals to obtain mice homozygous for the floxed *Eed* locus. βEzh2KO, βEed:βMll-DKO and Pdx1-EedKO were generated in similar fashion. By crossing animals harboring the YFP-reporter transgene to βEedKO animals, we generated lineage-tracing βEedKO-YFP mice. All mice had been backcrossed for over 10 generations before any phenotyping was initiated. Experimental mice were all males, unless otherwise stated. Age of the mice used for individual experiments are specified accordingly. Littermates of the same sex were randomly assigned to experimental groups.

Cell Line Model

Min6-B1 cell line were a kind gift from Dr. Philip Halban. Cells were cultured in DMEM supplemented with 15% fetal calf serum, 25 mM glucose, 71 μM 2-mercaptoethanol, 2 mM glutamine and 100 U/mL penicillin and 100 mg/mL streptomycin, in 5% CO_2 atmosphere incubator at 37°C.

Human Pancreatic Histological Samples

All human pancreas sections were obtained from the Network for Pancreatic Organ Donors with Diabetes (nPOD), a collaborative type 1 diabetes research project sponsored by the Juvenile Diabetes Research Foundation International (JDRF). Organ Procurement Organizations partnering with nPOD to provide research resources are listed at www.jdrfnpod.org/our-partners.php.

METHOD DETAILS

Animal Husbandry

All transgenic animals were maintained on a normal chow diet with 15% fat (Ssniff GmbH), fed ad libitum with free access to water (HCl acidified, pH 2.5-3) under controlled humidity and temperature with a 12-hour light and 12-hour dark cycle. Obese mice were fed with high fat diet (60% kcal% fat, Research Diet). All animal studies were performed with the approval of the local authority (Regierungspräsidium Freiburg, Germany) under license number 35.9185.81/G-10/94.

Mouse Embryo Isolation, Section Preparation and Staging

Female mice used for breeding were synchronized for their estrous cycle by exposing them bedding of mature male mice for 48 hr. After setting up the matings, vagina plugs were examined daily and the day of detection was designated E0.5. Pregnant mice were sacrificed on E14.5 and E17.5 using isofluorane and cervical dislocation. Embryos were dissected out of the uterus, washed in 4°C PBS and incised at the cervical region before overnight fixation in HistoFIX solution (CarlRoth) at 4°C. Embryos were then processed for paraffin embedding and 5um sagittal sections were collected on SuperFrost glass slides (Fisher). Hematoxylin-Eosin stainings were performed on some sections for confirmatory staging of the embryos. E14.5 embryos were identified for their digitally separated fingers, and absent eyelids. E17.5 embryos were identified for their wrinkled skin and long whiskers upon isolation, performed with reference to <<The Atlas of Mouse Development>>.

Glucose Tolerance Test and Plasma Insulin Measurements

For the oral glucose tolerance test (OGTT), mice were fasted overnight (16 hr), after which basal blood glucose was measured. Mice were given glucose (1 g/kg) by oral gavage. Blood glucose levels were measured using a OneTouch Vita blood glucose meter at 0, 15, 30, 45, and 60 min after glucose. Blood drawn using heparinized capillary tubes was centrifuged at 2000 *g* for 15 min at 4°C. Plasma obtained was used for insulin level measurement by ELISA (Mercodia Ultrasensitive Mouse Insulin Kit).

Islet Isolation

Adult pancreata were perfused through the common bile duct using a 27/30-gauge needle with Collegenase XI (Sigma) solution. Perfused pancreata were digested and purified by centrifugation in Histopaque gradient (Sigma). Isolated islets were hand picked and cultured in complete media (RMPI-1640 containing 11 mM glucose, 10% FBS, 0.1% Penicillin/Streptomycin) and maintained at 37°C in 5% CO_2 environment.

Histological Preparation of the Pancreas

Whole pancreata were dissected, weighed, washed in cold PBS and incubated for 24 hr at 4°C in HistoFix (CarlRoth). For β cell mass quantification, the pancreata were fixed in homemade cylindrical tube for form standardization of downstream quantification analysis. Each pancreas was then washed twice in PBS for 30 min each at room temperature, and dehydrated in 70% EtOH/dH_2O overnight, 95% EtOH/dH_2O for 3 hr and 100% EtOH for 3 hr at RT before being cleared with xylene for 15 min and embedded in paraffin. 5 μm sections were collected on SuperFrost glass slides (Fisher).

Immunofluorescence Stainings and Image Acquisition

Paraffinized sections (mouse and human) were prepared for immunofluorescence staining by heating the slides for 15 min at 55°C in an oven, deparaffinized (2 x 100% xylene 5 min each, 2 x 100% ethanol 5 min each, 2 x 95% ethanol 5 min each, 70% ethanol for 5 min) and rinsed in dH_2O for 5 min. Antigen retrieval was performed by heating the slides at 95°C for 20 min in HistoVT pH 7.0 (Nacalai USA) for all antibodies used. Specimens were blocked in 5% goat serum PBS-T for 15 min at RT before incubating with primary antibody diluted in 1% goat serum PBS-T overnight at 4°C. For primary antibodies produced in goat, donkey serum was used as the blocking agent. Each slide was rinsed three times in PBS, for 5 min each. Specimens were incubated in fluorochrome-conjugated secondary antibody diluted in 1% goat serum PBS-T for 1 hr at RT in the dark. After rinsing as above, VectorShield with DAPI and coverslip were mounted and slides were allowed to cure overnight at 4°C in the dark before image acquisition. For apoptosis assessment, we used DeadEnd Fluorometric TUNEL (Promega) and performed the staining as recommended by manufacturer. A list of antibodies used can be found in Key Resources Table.

Images were acquired using either ApoTome.2 (Zeiss) without structured illumination or LSM780 (Zeiss) and analyzed using ImageJ software unless otherwise stated.

Total RNA Extraction

Purified islets isolated were lysed directly in TRI reagent and total RNA was extracted according to the manufacturer's instructions. Briefly, cells were lysed in 1 mL TRI reagent and lysate was thoroughly mixed by pipetting. Samples in 1.5 mL tubes were incubated for 10 min at RT and 100 μL of 1-bromo-3-chloropropane was added. The mixture was shaken vigorously for 15 seconds and allowed to stand for another 15 min at RT. Tubes were centrifuged at 12,000 g for 15 min at 4°C and the aqueous phase was carefully transferred to fresh low-binding tubes (Eppendorf). 500 μL of 2-propanol and 1 μL of GlycoBlue Coprecipitant (Life Technologies) were added to the aqueous phase, mixed and incubated overnight at -20°C before centrifuging them at 12,000 g for 10 min at 4°C.

Supernatant was decanted and the RNA pellet washed twice with 1 mL 75% ethanol, air-dried and dissolved in 20 μL of RNase free water (Qiagen). RNA concentrations were quantified on a Qubit 2.0 Fluorometer (Life Technology).

Bulk RNA-seq and ChIP-seq

Total RNA extraction from purified islet is described above. mRNA from whole islets was used to generate libraries using Illumina TruSeq RNA Sample Prep v2 (RS-122-2001). The manufacturer's recommendations were followed and the libraries were sequenced on an Illumina HiSeq 2500 sequencer. All sequence data were performed in biological triplicates, each containing islets from 1-2 animals, at 2 x 50 bp length with high quality metrics (>20 Phred score) and nucleotide distribution. For 25 weeks βEedKO samples, two biological replicates were used, each containing islets from at least 4 animals.

NEXSON ChIP-seq workflow (Arrigoni et al., 2015) is applied to freshly isolated mouse islets. ChIP-seq performed using antibodies against H3K27me3 (Diagenode, #C15410195), H3K9me3 (Diagenode, #C15410193), H3K27ac (Diagenode, #C15410196), H3K4me3 (Diagenode, pAb-003-050), H2AK119ub (Cell signaling, #8240) and Pol-II (Diagenode, #C1520004). A list of ChIP-seq data sets used can be found in the Key Resources Table and Table S5.

Transcription Factor Over-Expression

Min6-B1 cell line were electroporated using Nucleofector II device (Lonza, Kit T, Program O-017) with pMAXGFP (Lonza) or mouse cDNA ORF clones of the 6 different transcription factors (Origene). 4 days after electroporation, cells were harvested, trypsinized and single cells were sorted in 384-well plates containing 240 nL of primer mix and 1.2 μL of PCR encapsulation barrier, Vapor-Lock (Qiagen GmbH, Germany).

Single Cell RNA-seq

Single cell RNA sequencing was performed using CEL-Seq2 method (Hashimshony et al., 2016) with several modifications. Importantly, a fivefold volume reduction was achieved using a nanoliter-scale pipetting robot, Mosquito HTS (TTP Labtech). Sorted plates were centrifuged at 2200 g for 10 min at 4°C, snap-froze in liquid nitrogen and stored at -80°C until processed. 160 nL of reverse transcription reaction mix and 2.2 μL of second strand reaction mix was used to convert RNA into cDNA. cDNA from 96-cells was pooled together before clean up and *in vitro* transcription, generating 4 libraries from one 384-well plate. 0.8 μL of AMPure/RNAClean XP beads (Beckman Coulter GmbH, Germany) per 1 μL of sample were used during all the purification steps including library cleanup.12 libraries (1152 single cells) were sequenced in a single lane (pair-end multiplexing run, 100 bp read length) of Illumina HiSeq 2500 sequencing system generating 200 million sequence fragments.

HDACi Administration

For HDACi experiment, SAHA was delivered through drinking water as previously described (Benito et al., 2015). Briefly, 0.67 g of SAHA was dissolved in 1 L of drinking water containing 18 g of β-cyclodextrin. The dose equates to 2 mg of SAHA per day for each animal. Mice were treated for 12 weeks from 8 weeks of age before OGTT examination.

QUANTIFICATION AND STATISTICAL ANALYSIS

Bulk RNA-seq Bioinformatic Analysis

Reads for mouse bulk RNA-seq datasets were mapped with TopHat v2.0.13 against mouse genome version GRCm38. The total number of sequenced reads ranged from 10-50 million pairs of which at least 68% of the reads were mapped. Reads were counted with featureCounts (subread-1.5.0-p1) against gene models from Gencode version M9. Differential expression analysis was performed with edgeR (v3.14). Alignment statistics (data not shown) indicated data were of high quality and sequencing depth was sufficient to test for differential expression between conditions. Isoform-level analysis was performed with Isolator (www.biorxiv.org/content/early/2016/11/20/088765). For human dataset, DESeq2 package v1.8.1 was used for differential gene expression in diabetic patient samples. The count matrix was used as given (GEO: GSE50244). Differential genes were called with an FDR threshold of 0.05. The mapping from mouse to human orthologues was done with Biomart (http://www.ensembl.org/biomart). Correlations between mouse and human expression fold changes were calculated in R using Pearson correlation.

Single Cell RNA-seq Analysis

Read 2 of the each read pair was first 3' trimmed for adapters, base quality and poly-A tails using cutadapt v1.9.1. Remaining reads were mapped with STAR v2.5.3.a against mouse genome version GRCm38 with gene models from Gencode version M9. Gene summarization was done using featureCounts (subread-1.5.0-p1) collapsing exons to genes. Genes with a biotype related to pseudogenes as well as multimapping reads were discarded. Cell demultiplexing was done using the cell-barcode and the unique molecular identifier (UMI) present in the first 12 nt of Read 2 of the read pair.

Data analysis was performed using RaceID2 and StemID algorithm (Grün et al., 2016). Downsampling to 5,000 transcripts was used for data normalization. K-medoids clustering was performed using 1- Spearman's correlation as a distance metric. The minimum suitable cluster number (=4) characterizing the dataset was determined by computing Jaccard's similarity for each cluster by bootstrapping for k-medoids clustering with different cluster numbers. The minimum number yielding a Jaccard's similarity >0.6 for all clusters was selected. The t-distributed stochastic neighbor embedding (t-SNE) algorithm was used for dimensional reduction and

cell cluster visualization. Since outlier identification is beyond the scope of the paper, RaceID2 was executed with the probability threshold value for outlier identification set to zero. The StemID algorithm was used to infer a dedifferentiation trajectory. A p value threshold of 0.05 was chosen to assign significance to the links. StemID identified a dedifferentiation trajectory along the clusters 1-2-3-4.

To identify modules of co-expressed genes along the dedifferentiation trajectory, all cells assigned to these links were assembled in a pseudo-temporal order based on their projection coordinate. All genes that were not present with at least three transcripts in at least a single cell were discarded from the sub-sequent analysis. The pseudo-temporal gene expression profiles of all genes were subsequently z-score transformed and topologically ordered by computing a one-dimensional self-organizing map (SOM) with 1,000 nodes. We then defined modules of co-expressed genes by grouping neighboring nodes of the SOM if average gene expression profiles at these nodes exhibit a Pearson's correlation coefficient larger than 0.9. Only modules with more than 5 assigned profiles were retained for visualization of co-expressed genes.

Gene-Set Enrichment Analysis

For gene-set enrichment analysis, javaGSEA desktop application from Broad institute was used. Cufflink transcriptome profile for each sample was used without any data pre-filtering. Pair-wise gene-set enrichment analysis was performed using Gene Ontology (GO) gene set collection derived from gene2go annotation data from NCBI with the latest update (April_2015), c2cp and manually curated gene sets from (Xie et al., 2013) and (Blum et al., 2012). Number of permutations were set to default 1000 and permutation type set to "gene_set" and chip platform set to "Gene_Symbol.chip". Enrichment statistics, metric for ranking genes, gene list sort and ordering mode were kept at default values.

ChIP-Sequencing Bioinformatics Analysis

All mouse ChIP-seq data (our data as well as external data) were mapped with Bowtie2 (v2.2.8) and filtered for PCR duplicates. Bigwig tracks for visualization in IGV were created with DeepTools v2.4.1 and normalized to sequencing depth (Ramírez et al., 2016). For Figures 5F and 5G, H3K27ac and H3K4me3 peaks were called in "broad" mode in MACS2 (v2.1.1.20160309). Histone breadth is defined as peak length from the start to the end of each called peak regions. For Figure 5E area under curve was calculated as sequencing-depth normalized ChIP-seq signal. A list of ChIP-seq data sets used can be found in Table S5.

Chromatin Segmentation

We used EpicSeg for chromatin segmentation (Mammana and Chung, 2015). We combined our own ChIP-seq data for H3K4me3, H3K27ac, H3K27me3, H3K27me2, H3K27me1, H3K36me3, H2AK119Ub, H3K9me3 and Pol2 together with external available data for H3K4me1, H3K27me3 and H3K9me3 marks (Hoffman et al., 2010; Tennant et al., 2013). EpicSeg uniquely assigns reads or fragments to genomic bins (size 200 bp) and hence is able to combine single and paired end data. Replicates are averaged. In addition we incorporated methylation data from young islets (Avrahami et al., 2015) by adding an additional column to the internal matrix of EpicSeg. For this purpose we calculated the median methylation in a 600 bp window and a shift size of 200 bp. The binned methylation percentages were then inversely scaled. By this transformation, most of the genomic bins for methylation have low or 0 values (ie. high methylation) whereas regions with a lower methylation have higher values. Chromatin states were assigned to genes according to (1) the maximum single state coverage over the genebody (genebody state) and (2) the chromatin state at the TSS (TSS state). Assignments were only done to the "basic" subset from Gencode M9 and to genes with biotype "protein_coding","lincRNA" or "antisense". Of note, original methylation data (GEO: GSE68618) from young islets (Avrahami et al., 2015) was remapped to GRCm38 with bwa-meth and methylation levels were obtained with MethylDackel/PileOMeth (https://github.com/dpryan79/MethylDackel). Chromatin segmentation and integrated expression analysis was only done on autosomes (1-19) as some of the external ChIP-seq data came from female mice, whereas for our own data we used only male mice.

Image Quantifications

For all morphometric analysis of islets, 4-5 animals of each genotype were analyzed. Each section was stained for insulin (β cells), glucagon (and pancreatic polypeptide and somatostatin) and/or Chromogranin A (total islet area) and DAPI (cell count and total pancreas area estimation). For β cell mass analysis, 4 sections ~200 μm apart were covered systematically by accumulating images from non-overlapping fields with ApoTome.2 (Zeiss) using a 10X objective for whole pancreas section. For other endocrine cell area analysis, 20-50 islet profiles were chosen randomly from each sections and captured using a 40X objective. All morphometric analyses were performed using ImageJ software. Briefly, individual channels were converted to 8-bit grayscale and measurement scale was converted from pixels to μm. A identical threshold was applied to all images from the same channel to exclude background signals and further converted to binary format before automated analysis of immunoreactive area. β cell area was expressed as a fraction of total surveyed pancreatic area and β cell mass was estimated by multiplying the fraction to pancreas weight. Other endocrine cell area was expressed as a fraction of total islet area surveyed by total islet area from Chromogranin A immunoreactivity. β cell size was estimated by dividing the total β cell area by the total number of β cell nuclei that it contained. For all analysis, the region of interest was traced carefully to exclude other tissues such as ducts, lymph nodes, and mesenteric tissues.

For intra-islet quantification, 15-30 islets were chosen randomly from at least 2 sections spaced ~200 μm apart for each animal. 4-5 animals were used per genotype. For mouse samples, H3K27me3 positivity and intensity were assessed using Image J measurement functions in a blinded fashion. For human samples, analysis of H3K27me3, H3, Insulin and DAPI intensities were automated using customized Cell Profiler pipeline (available upon request).

Statistical Analysis

All data unless otherwise stated are shown as mean value ± standard error of the mean (SEM) and tested statistically using two-tailed Student's t test or ANOVA. All figures and statistically analyses were generated using GraphPad Prism 7. $p<0.05$ was considered to indicate statistical significance.

DATA AND SOFTWARE AVAILABILITY

The accession number for the RNA-seq data reported in this paper is GSE110648.

Cell Metabolism, Volume *27*

Supplemental Information

The Polycomb-Dependent Epigenome Controls β Cell Dysfunction, Dedifferentiation, and Diabetes

Tess Tsai-Hsiu Lu, Steffen Heyne, Erez Dror, Eduard Casas, Laura Leonhardt, Thorina Boenke, Chih-Hsiang Yang, Sagar, Laura Arrigoni, Kevin Dalgaard, Raffaele Teperino, Lennart Enders, Madhan Selvaraj, Marius Ruf, Sunil J. Raja, Huafeng Xie, Ulrike Boenisch, Stuart H. Orkin, Francis C. Lynn, Brad G. Hoffman, Dominic Grün, Tanya Vavouri, Adelheid M. Lempradl, and J. Andrew Pospisilik

Figure S1 (Related to Figure 1)

A

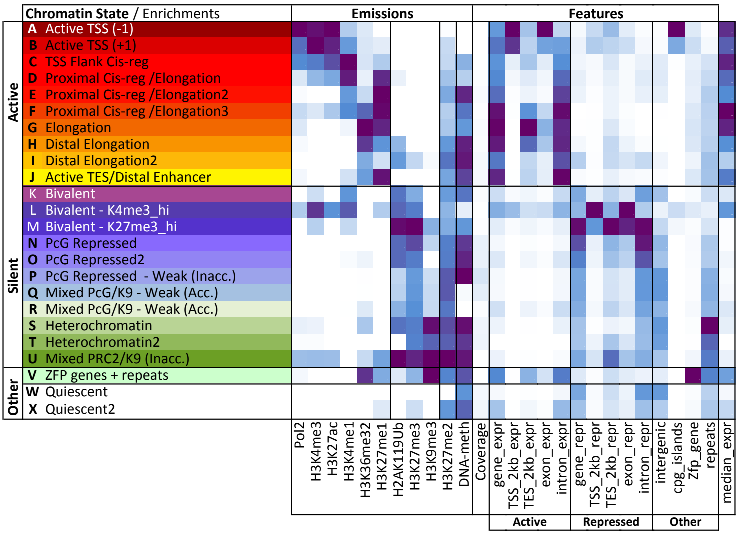

B

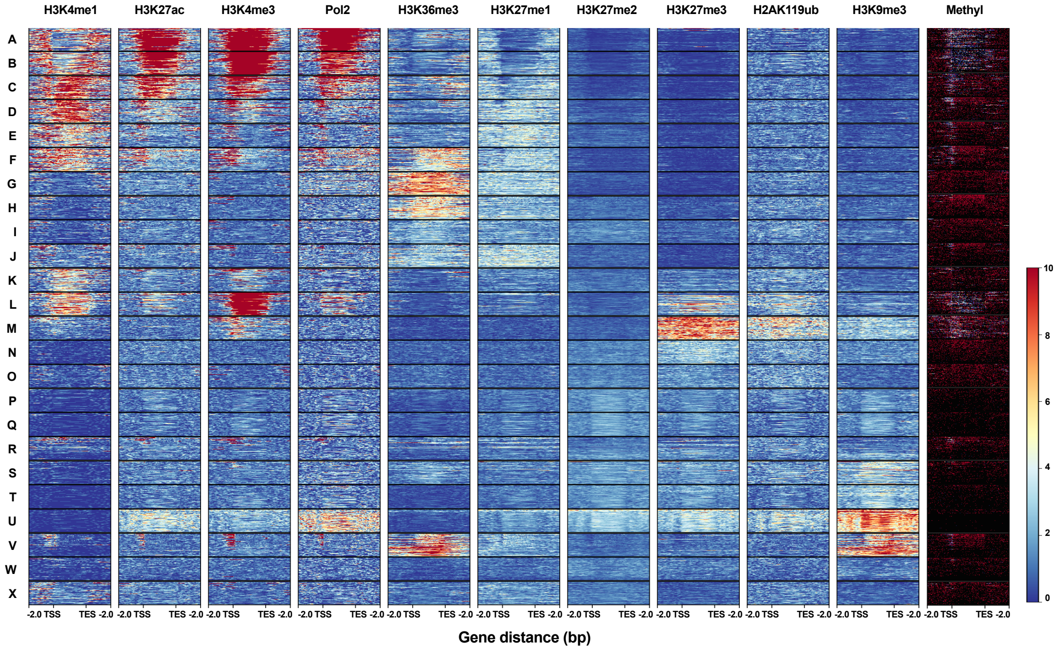

C

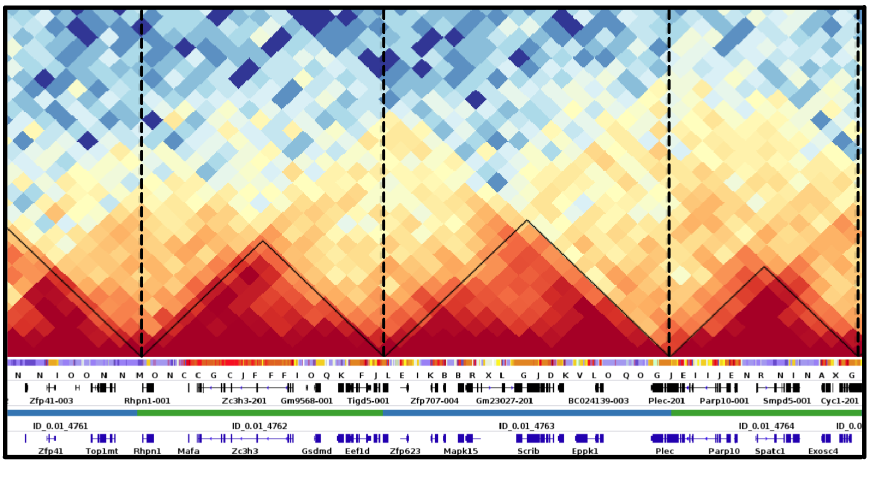

Supplementary Figure S1.

A.Histone mark characteristics, gene expression levels and genomic features of healthy islets. EpiCSeq chromatin state segmentation and manual annotation according to emissions and annotations in D. (Inacc=Inaccessible; +1 or -1=± nucleosome position around TSS, expr = expressed, repr= repressed, median_expr= Median expression)

B.Heatmap profiles of average normalized read coverage from ChIP-seq signals for various histone marks, Pol2 and CpG methylation signal of 100 genes from each chromatin state.

C.Example region showing Hi-C TAD boundaries aligning with chromatin state segmentation boundaries. Dash lines indicates TAD boundaries. (Hi-C image taken from http://chorogenome.ie-freiburg.mpg.de; dataset mouse CH12; GEO accession GSE63525)

Figure S2 (Related to Figure 2)

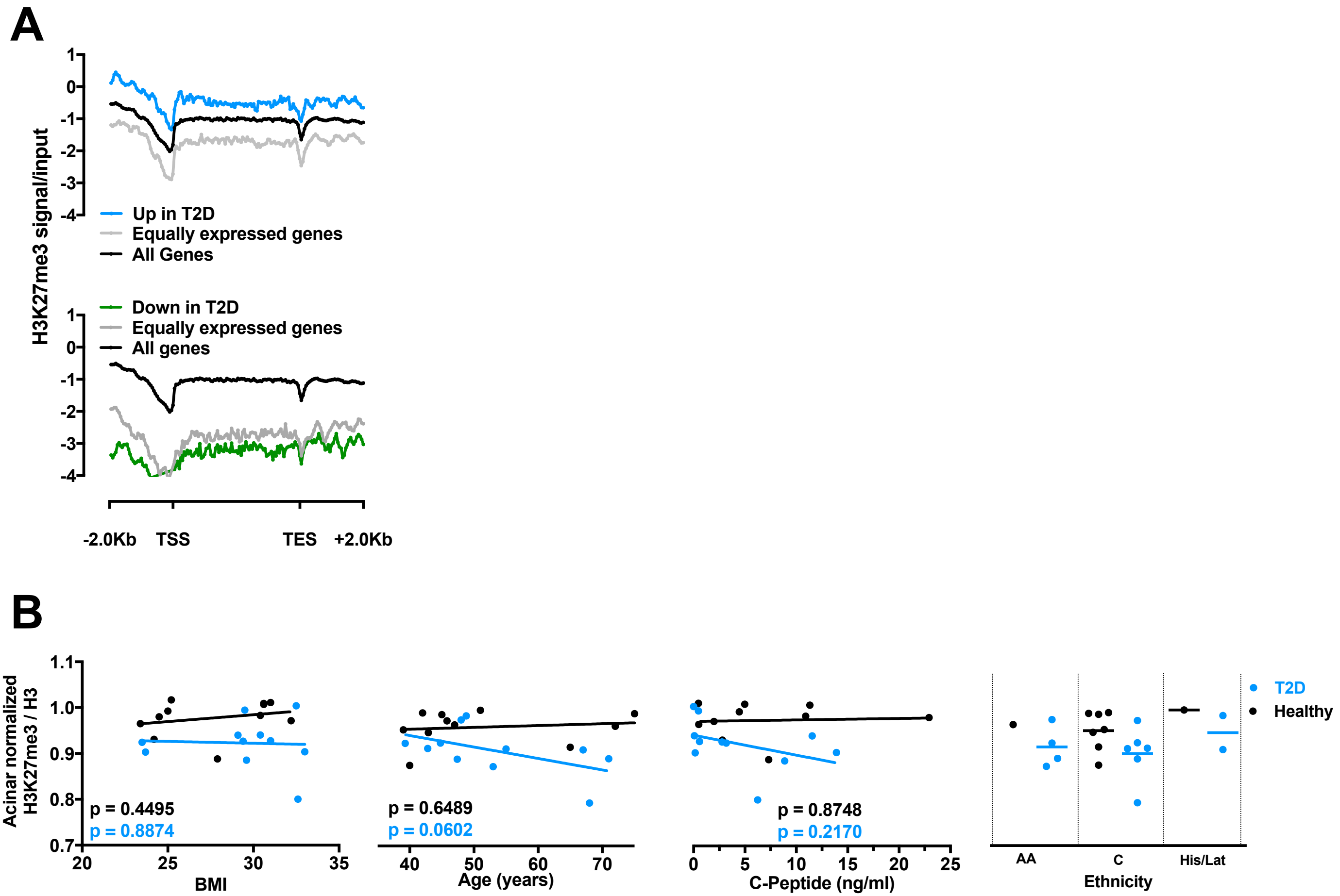

Supplementary Figure S2.

A. Average H3K27me3 signals at genes up or down-regulated in T2D and equally expressed genes. TSS: transcription start site, TES: transcription end.

B. H3K27me3 immunostaining intensity in β-cells of pancreatic sections from T2D healthy (black) and diabetic (blue) donors vs BMI, age, C-peptide levels or ethnicity. Solid lines = linear regression analysis of groups. $P < 0.05$ indicates regression slopes being significantly non-zero.

Supplementary Figure 3 (Related to Figure 3)

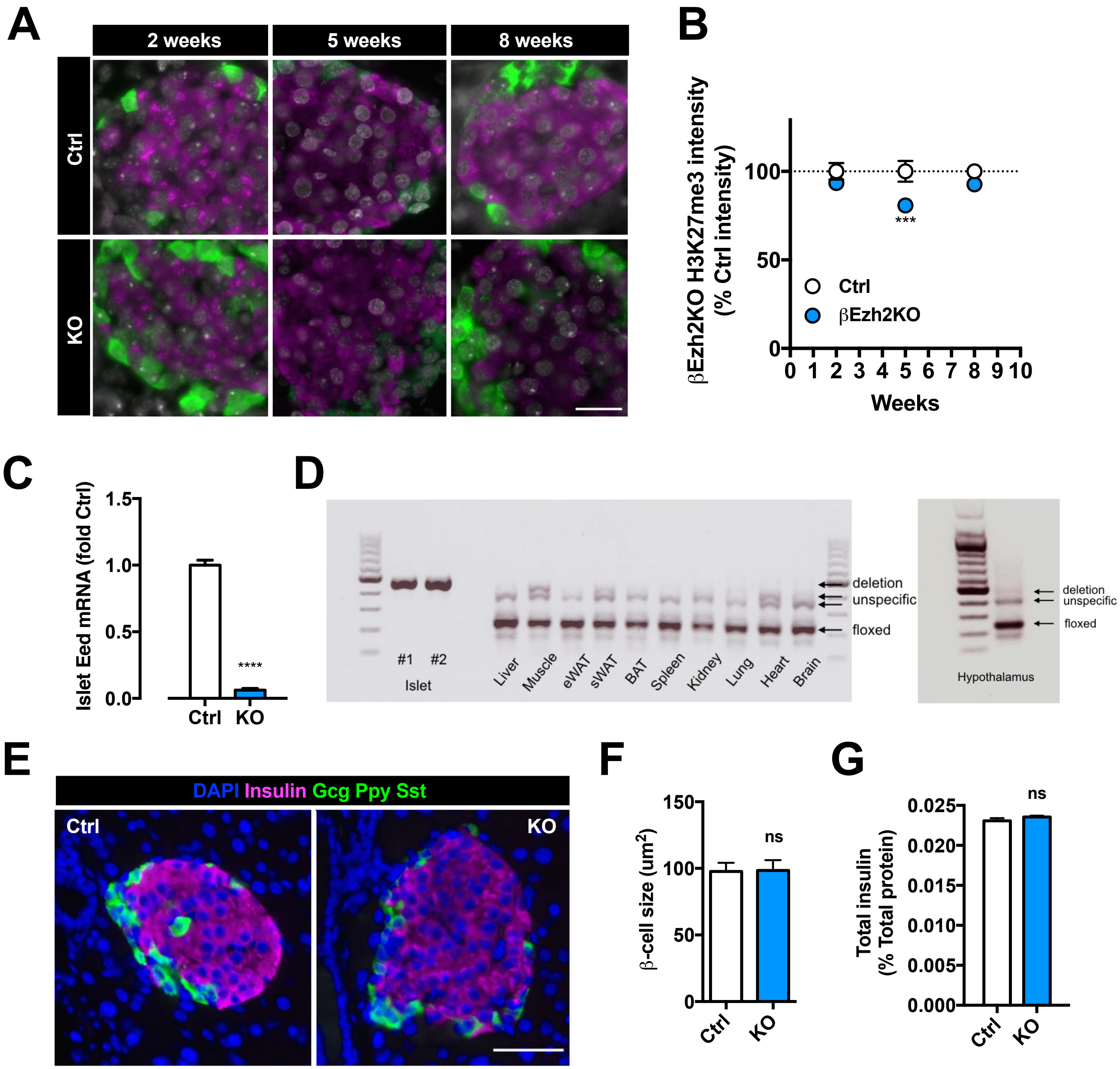

Supplementary Figure S3

A.Representative images of immunostaining for H3K27me3 (grey), insulin (magenta) and glucagon (green) in Ctrl (top) and βEzh2KO (bottom) islets at 2, 5 and 8 weeks of age.

B.Quantification of relative intensity of H3K27me3 immunostainings of βEedKO and control islet β-cells at different ages. All data points were normalized to alpha-cell H3K27me3 levels in the same islets prior to comparison.

C.Quantitative RT-PCR showing loss of Eed mRNA in βEedKO islets.

D.PCR validation of *Eed* allele deletion in islets and other tissues of βEedKO animals. One animal is represented here for all tissues except for islets where two animals are shown

E.Immunofluorescence staining of islet hormones in Ctrl and βEedKO islets at 8 weeks of age.

F.-G. Quantification of total β-cell mass (left) and insulin area (right) per islet shows no significant difference between Ctrl and βEedKO animals at 8 weeks of age.

Data represents mean ± SEM. Scale bar = 25uM. *** $p<0.001$, **** $p<0.0001$, ns = $p>0.05$.

Supplementary Figure 4 (Related to Figure 4)

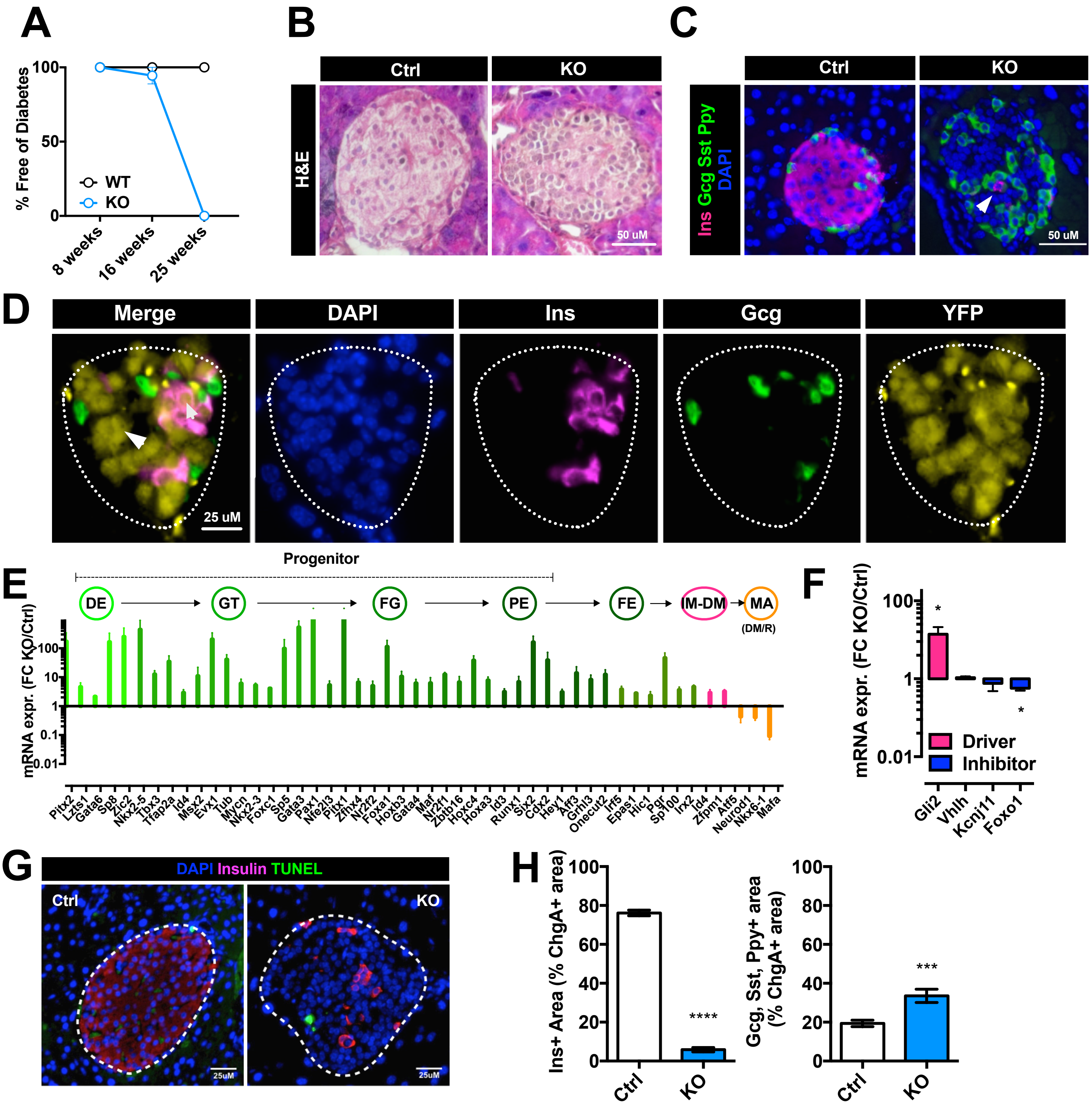

Supplementary Figure S4.

A.Percentage of diabetes-free individuals at 8, 16 and 25 weeks of age. Diabetes was defined as blood glucose >300mg/dL after 2 hours fasting. Note that all animals developed diabetes at 25 weeks of age

B.H&E staining of pancreatic sections showing readily detectable islets and reduced inter-nuclei spacing in βEedKO mice

C.Immunofluorescence stainings for hormones of ctrl and βEedKO islets. White arrow depicts cell with residual insulin staining.

D.Representative image of βEedKO islets harbouring a RIP-cre inducible YFP lineage tracer (yellow) stained for insulin (magenta) and glucagon (green). Small white arrow indicates insulin and YFP positive β-cell, large white arrow indicates insulin negative YFP positive cell. White dotted line outlines the islet.

E.Mean expression fold change of transcription factors in mRNA-seq data from βEedKO and Ctrl islets (Gene sets from DE: definitive endoderm, GT: gut tube, FG: fore gut, PE: pancreatic endoderm; Xie et al., 2013) and immature β-cells (IM-DM; Blum et al., 2012, MA: REACTOME: *Regulation of Gene Expression in Beta-Cell*). Arrow represent hypothetical developmental trajectory. Genes listed show core enrichment in GSEA analysis and are significantly different between healthy and T2D islets with FDR-q<0.05.

F.Mean expression fold change of known factors implicated in β-cell de-differentiation in mRNA-seq data from βEedKO and Ctrl islets. * FDR-q<0.05

G.Representative images of TUNEL treated Ctrl and βEedKO pancreas. White dotted line indicates the border of islets

H.Morphometric analysis of immunostaining: % β-cell area (Ins+, left panel) and α- (Gcg+) , δ- (Sst+) and PP-cell (Ppy+) area (right panel) within the endocrine compartments (ChgA positive) of islets at 25 weeks of age. *** p<0.001, **** p<0.0001

Figure S5 (Related to Figure 5)

A

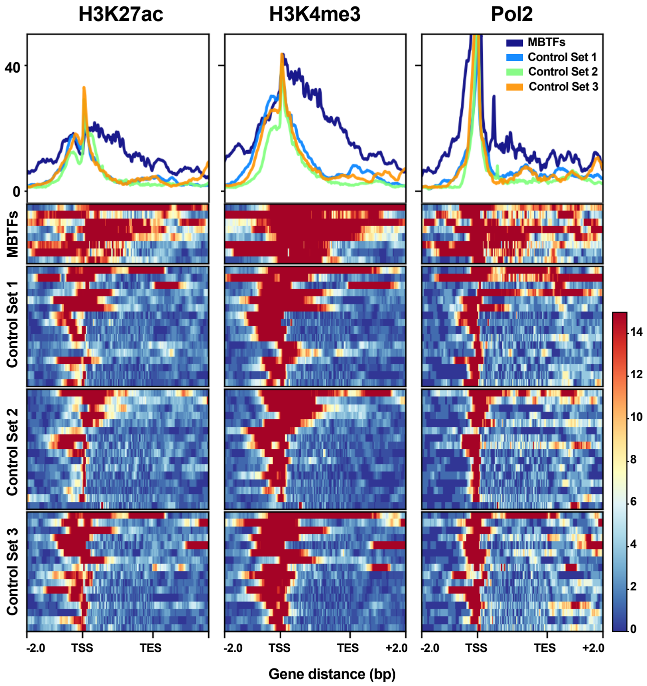

B

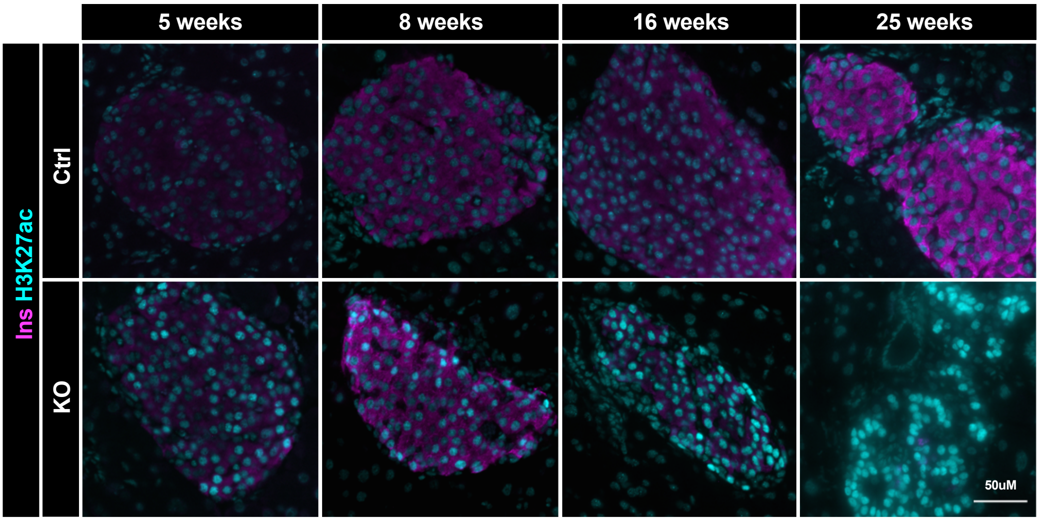

C

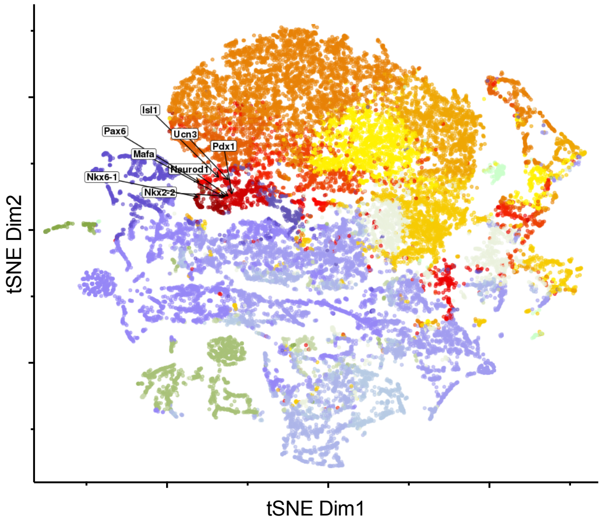

Supplementary Figure S5.

A.Profiles of average normalized read coverage from ChIP-seq signals for H3K27ac, H3K4me3 and PolII for MβTFs and control gene sets of similar gene expression in mouse β-cells (top). Profiles are presented as heatmaps and sorted according to H3K4me3 signals (bottom).

B.Representative immunostaining images for H3K27ac (cyan) and insulin (magenta) in Ctrl and βEedKO islets at 5, 8, 16, and 25 weeks of age.

C.tSNE representation of all genes colored according to chromatin state (color coded based on Figure 1E). MβTFs are highlighted.
